# Cognitive and neuropsychological correlates of the attention training technique: a systematic review and evidence synthesis

**DOI:** 10.3389/fpsyt.2026.1766748

**Published:** 2026-06-09

**Authors:** Clair Davison, Lora Capobianco, Karin Carter, Adrian Wells

**Affiliations:** 1 Division of Psychology and Mental Health, School of Psychological Sciences, Faculty of Biology, Medicine and Health, The University of Manchester, Manchester, United Kingdom; 2 Research and Innovation, Greater Manchester Mental Health National Health Service (NHS) Foundation Trust, Manchester, United Kingdom; 3 NHS Trafford Talking Therapies Service, Altrincham Health and Wellbeing Centre, Manchester, United Kingdom

**Keywords:** attention flexibility, attention training technique, cognitive correlates, executive control, mechanisms, metacognition, neurophysiology

## Abstract

**Introduction:**

The Attention Training Technique (ATT) is a brief metacognitive intervention recognised as a possibly efficacious standalone transdiagnostic treatment for emotional disorders. The cognitive and neuropsychological mechanisms underlying its clinical effects are of particular interest in understanding and developing the technique. The aim of the systematic review was to synthesise and evaluate the cognitive-attentional task performance and neurocognitive correlates of ATT in the context of theoretical mechanisms from which ATT is derived.

**Methods:**

Five electronic databases (PsycINFO, MEDLINE, PubMed, Web of Science and EMBASE) were searched from January 1990 to November 2025. Studies that used ATT as part of a metacognitive multi-component treatment package or combined with other therapy/technique(s) were excluded. Sample inclusion was diverse to capture effects on non-clinical and clinical individuals and across age groups for potential sub-group analyses.

**Results:**

In total, 20 studies with 1, 230 participants met the inclusion criteria. Four studies included clinical samples, four studies included non-clinical participants, two studies used experimental induction of pain or mind wandering, and 10 used healthy samples of which two used school children. Study quality varied from strong to weak with the majority receiving ‘moderate’ ratings. Across 14 cognitive-attentional tasks and three neural methodologies (EEG, fNIRS, fMRI), the review found small to large cognitive and neural effects associated with ATT. Nine cognitive tasks showed significant ATT-dependent effects in at least one study, with the most consistency shown on the emotional dot-probe. Neural findings across all methodologies converged, suggesting that ATT modulates cognitive control, frontoparietal, dorsal attention networks and reduces default mode network connectivity.

**Discussion:**

Interpretation and synthesis of findings based on the S-REF model are consistent with cognitive and neural effects involving reduced threat monitoring, improved executive control, and enhanced disengagement from self-referential processing; central theoretical mechanisms and design parameters of ATT. Where inconsistencies across study effects emerged, they may be due to heterogeneity in cognitive task and measurement factors and ATT protocol deviations. Future research on individual differences in neurocognitive effects associated with ATT across clinical and sub-clinical populations is needed. Studies must safeguard fidelity and adherence to the ATT protocol and improve reporting of these important factors.

**Systematic review registration:**

https://www.crd.york.ac.uk/prospero/, identifier CRD42024483053.

## 1 Introduction

The Attention Training Technique (ATT) is a brief auditory-based metacognitive treatment method designed by Wells (1) consisting of a series of exercises involving externally-focused, selective attention, attention switching and divided attention exercises. The structure and content of the technique was designed to reduce self-focused modes of perseverative processing, reduce threat monitoring and enhance higher-order metacognitive control of cognition (2, 3).

Although originally intended to be used as part of Metacognitive Therapy (MCT) (4), when evaluated on its own, ATT was found to be associated with reductions in clinical symptoms across a range of diagnoses (e.g., 1, 5–8). Such early findings prompted further research into the efficacy of ATT as a standalone treatment technique. Fergus and Bardeen (9) reviewed 15 treatment studies across clinical and subclinical samples experiencing emotional difficulties. They concluded ATT to be a beneficial transdiagnostic intervention, yielding moderate-to-large therapeutic effects, and one which overcomes challenges of disorder-specific approaches in the context of dealing with comorbidities. However, they argued that robust conclusions about its efficacy as a standalone technique proved difficult as it was often evaluated in conjunction with, or as an adjunct to other therapeutic treatments/techniques. Knowles and colleagues (10) conducted a systematic review of 10 studies confining their analysis to standalone ATT studies across both clinical (*n* = 6) and non-clinical (*n* = 4) samples. They concluded that preliminary evidence exists across single-case trials and small randomised controlled trials in support of ATT as an effective standalone treatment for emotional disorders, with encouraging levels of symptom reduction and diagnostic remission. They also highlighted some evidence for ATT being possibly efficacious in the treatment of positive symptoms in schizophrenia, a finding more recently shown in a single case alternating-treatments study in schizo-affective disorder (11).

ATT is grounded in the Self-Regulatory Executive Function (S-REF) (12, 13) model of psychological disorders, also known as the metacognitive model. According to the model, emotional disorder is caused by biased knowledge, control functions and strategies involved in the top-down regulation of thinking. A fundamental principle is that disorder is maintained by a universal thinking style, the Cognitive Attentional Syndrome (CAS), characterised by perseverative negative processing in the form of worry and rumination, attentional monitoring for threat and maladaptive coping strategies (e.g., avoidance, thought-suppression, inactivity). A surface marker and component of the CAS is excessive and inflexible self-focused attention. Individuals are psychologically vulnerable to the CAS because underlying biases in metacognition (e.g., declarative and procedural knowledge and control processes) activate and maintain perseverative responses to negative mental events, including intrusive thoughts and discrepancies in self-regulation. For example, metacognitive knowledge that worrying and/or rumination are uncontrollable reduces the implementation of adaptive internal control and reinforces the CAS by adding negative interpretation of thinking to thinking itself, extending the ruminative process and increasing subjective threat (3).

ATT was developed to counteract the CAS and modify causal mechanisms represented in the S-REF model. This includes interrupting perseverative self-focused processing (e.g., worry and rumination), improving flexible attentional and executive control, and reinforcing adaptive metacognitive knowledge. As such, the impact of ATT should be detectable in cognitive and neural task paradigms that are sensitive to these effects. The S-REF model can be used as a framework for conceptualising findings from a diverse range of paradigms if we can map different cognitive-task and neural effects onto important processes and structures of the SREF model. In the present synthesis we examined whether task and neural correlates of ATT were consistent with theoretically plausible changes within three parts of the S-REF model; i) reduced threat monitoring; ii) enhanced executive control/regulation; iii) reduced internally-focused CAS/thinking strategies, by specifying clusters of tasks/paradigms indicative of these parts.

For example, consistent with Wells and Matthews (12), we view laboratory tasks of attention such as the emotional dot-probe and emotional Stroop to be indicative of threat-monitoring (sustained and inflexible focus on danger) in the S-REF. Therefore, such tasks of attention disengagement from threat/emotion are relevant to this dimension. Further justification for our mapping stems from changes in the CAS (worry, rumination) and attentional flexibility as potential mechanisms of ATT based on self-report changes in attentional control (14, 15) and objective attentional and neural correlates (16–18). Whilst ATT has been associated with improved performance across a range of laboratory-based tasks, including versions of the Stroop and dot-probe (19–23), inconsistencies appear to exist (24, 25). Preliminary evidence suggests ATT might work by training frontoparietal regions associated with the Cognitive Control Network (CCN; 26) and Dorsal Attention Network (DAN; 27) (28, 29); and perhaps, influences neural connectivity differently depending on level of the CAS (30). However, such findings are yet to be systematically reviewed, synthesised, critically evaluated and conceptually mapped onto the S-REF framework as specified. Establishing whether the attention-task and neuropsychological changes associated with ATT are consistent with the mechanisms identified within the S-REF model is an important step in assessing ATT’s applicability and precision as a targeted treatment technique (31–33). The specific clusters of cognitive and neuropsychological findings relevant to each part of the S-REF are described later in the conceptual synthesis section of the paper.

### 1.1 Brief outline of ATT

ATT is an auditory *external attention* exercise, lasting approximately 12-minutes, that guides the listener to attend to an array of parallel and non-parallel sounds of different loudness at different spatial locations. There are three attention and control components, practised in series in the 12-minute procedure: selective attention, rapid attentional switching and divided attention (see Wells (2) for a detailed description). The first two components last approximately 5 minutes each and divided attention approximately 2 minutes. The task is configured to be attentionally demanding by including auditory stimuli in parallel and by increasing the speed of attention switching gradually during the switching phase. When used in therapy, ATT is usually embedded in a rationale and a meta-level dialogue, where the therapist explores metacognitive beliefs and shape a person’s experiences of cognitive control. However, the use of a rationale and meta-level dialogue varies and is not always present in laboratory studies that have tested individual components of the technique.

### 1.2 Current review

This review is the first, to our knowledge, to systematically review together both the objective cognitive-attentional task performance and neurocognitive correlates associated with ATT. The review explored if there are specific or widespread effects, whether they are consistent across laboratory-based cognitive attentional tasks and neural measures. To interpret and synthesise findings we meaningfully mapped cognitive and neural paradigms onto three S-REF areas: i) threat monitoring, ii) executive control/regulation, iii) internally-focused CAS/thinking strategies.

## 2 Methods

The Preferred Reporting Items for Systematic Reviews and Meta-Analyses (PRISMA) (34) guidelines were followed throughout the development and completion of this review. The protocol was registered with the International Prospective Register of Systematic Reviews (PROSPERO) in January 2024 (ID CRD42024483053).

### 2.1 Search strategy

The search strategy was developed through discussion between the authors and in consultation with the University of Manchester Academic Library (**Table 1**). Search terms were categorised according to each component of the research question and aimed to capture the range of cognitive processes relevant to the S-REF model and measures used to evaluate them. Categories included: *ATT (as developed by Adrian Wells)*, *Cognition*, *Neural Measures*, *Cognitive Tasks*, *Psychological Morbidity*, and *Population*. Medical Subject Headings (MeSH) and free-text terms were used to identify synonyms. Boolean operators (“AND”, ‘OR”) were used to combine terms and concepts. A systematic search for peer-reviewed articles published between the year 1990 to November 2025 was conducted using five electronic databases: PsycINFO, MEDLINE, PubMed, Web of Science and EMBASE. A start date of 1990 was chosen because this was the year of the first ATT publication (1). Supplementary searching including forwards and backwards citation chasing, specific author searches, and handsearching of key related reviews was also completed.

**Table 1:** Search strategy.

#1	“Attention Training Technique” or “Adrian Wells”
#2	“Cogniti*” or “Cognitive Processes” or “Executive Processes” or “Higher Order Processes” or “Executive Function” or “Executive Control” or “Cognitive Control” or “Attention*” or “Attention* Bias” or “Attentional Awareness” or “Attentional Set Shifting” or “Self-focused Attention” or “Divided Attention” or “Focused Attention” or “Sustained Attention” or “Selective Attention” or “Present-Focused Attention” or “Internally focused Attention” or “Externally focused Attention” or “Attentional Switching” or “Attentional Flexibility” or “Attentional Shifting” or “Attentional Drifting” or “Attentional Performance” or “Cognitive Flexibility” or “Attentional Disengagement” or “Task Switching” or “Inhibition” or “Inhibitory Control” or “Set-Shifting” or “Hypervigilance” or “Distractions” or “Delay of Gratification”
#3	“Neurop*” or “Neurocognitive” or “Neuropsychology” or “Frontoparietal” or “Neuroimaging” or “Functional Magnetic Resonance Imaging” or “fMRI” or “Functional Near Infrared Spectroscopy” or “fNIRS” or “Electroencephalography” or “EEG” or “Magnetic Resonance Imaging”
#4	“Dot Probe” or “Emotional Stroop” or “Colour Word Stroop” or “Day/Night Stroop” or “Cognitive Battery” or “Cognitive Test” or “Attentional Network Task” or “2-Back 3-Back” or “Dichotic Listening” or “ACCE” or “Trail Making” or “Digit Span” or “Text Comprehension” or “Story learning” or “Digit Ordination of Rey” or “Word-List Learning” or “Test of Attention” or “Neuropsychological Battery” or “Neuropsychological Assessment”
#5	“Depress*” or “Anxi*” or “Mood” or “Feeling” or “Symptoms” or “Pain” or “Traumatic stress” or “Rumination” or “Worry”
#6	“Adults” or “Children” or “Students” or “Undergraduates” or “Healthy” or “Non-Clinical” or “Clinical”
#7	#2 OR #3 OR #4 OR #5 OR #6
#8	#1 AND #7
#9	*Limit to Publication Year 1990-2025*

### 2.2 Study selection

Identified references were imported into the software Rayyan (35) prior to deduplication and screening. Duplicates were identified and removed by the software. The first author screened the titles and abstracts of each article according to pre-specified inclusion and exclusion criteria before screening the full texts of remaining studies. An independent reviewer also screened the titles and abstracts of all identified studies (*n* = 169). Agreement was moderate (Cohen’s kappa (*κ*)= 0.53). Discrepancies arose from one reviewer being more cautious with instances of uncertainty resulting in 17% more studies being screened for inclusion for full text review by one reviewer compared to the other. A second independent reviewer also screened the full texts of a random 20% of the sample (*n* = 11) which yielded full inter-rater agreement (*κ* = 1.0). Instances of uncertainty at the full-text review stage were resolved through discussion with the research team.

### 2.3 Inclusion and exclusion criteria

The Population, Intervention, Comparator, Outcome, Study Design (PICOS) framework (36) was used to inform eligibility criteria (**Table 2**). The study was not restricted by non-clinical, clinical, diagnostic or demographic status of participants or study task parameters in keeping with a comprehensive analysis of ATT effects and allowing unbiased capture and summarising of findings that might support potential sub-group meta-analyses and detailed theoretical synthesis. As ATT is a general technique that is potentially relevant to universal mechanisms cutting across normality, abnormality, age and diagnoses, there were no exceptions made to the populations that could be included in the review (e.g., children, adults, clinical and non-clinical populations across all diagnostic categories, including comorbidities) important for revealing any sub-group variation in findings and to inform generality of effects. All versions of Adrian Wells’ ATT as a standalone intervention, including modified or translated version were included. Studies were only included if they were written in English or Italian, the fluent languages of the research team. Studies were excluded if they were published before 1990 or used ATT as part of the metacognitive multi-treatment package or with other therapy/techniques(s). Grey literature, including conference abstracts, papers, dissertations or theses were also not included.

**Table 2:** Inclusion and exclusion criteria using the PICOS framework.

Parameter	Inclusion	Exclusion
Population	Adult (18+) or Child (0-17); Clinical or Non-Clinical across multiple diagnostic categories (with nil excluded diagnoses).	–
Intervention	Adrian Wells’ ATT (1, 2) as a standalone intervention, including modified or translated versions.	Studies published before 1990. Studies using ATT within the metacognitive therapy treatment package or with another therapy/technique.
Comparator	Baseline; active control groups; no intervention control groups, standard care and waiting lists.	–
Outcome	Relationship between ATT and objective cognitive attentional task performance and/or neurophysiology.	–
Study Design	Peer-reviewed interventional studies (including but not limited to randomised controlled trials, randomised experimental designs, quasi-experimental, case-controlled, pre- and post, SCEDs and case studies). There were no restrictions on settings used.	Grey literature, including conference abstracts, papers, dissertations or theses.

### 2.4 Data extraction

The first author independently extracted data from studies meeting full inclusion criteria using a standardised data extraction sheet. This included general study information (e.g., author/year, country, title DOI), study characteristics (e.g., aims/hypotheses, study design), sample characteristics (e.g., age, gender, non-clinical or clinical descriptors), intervention (e.g., type, delivery mode, length, number and frequency of sessions), primary and secondary outcomes and key findings (including means, standard deviations and effect sizes, where available). The data extraction was verified by a second reviewer for accuracy and completeness. Authors of studies were contacted to provide missing or additional data where there was insufficient information to extract or compute relevant data. In preparation for synthesis, studies were grouped according to (1) whether they evaluated ATT using an objective cognitive attentional measure, neural measure or both and (2) their functional relationship with the causal mechanisms identified in the S-REF model using subcategories of threat monitoring, executive control/regulation and internally focused CAS/thinking strategies.

### 2.5 Quality assessment

The Effective Public Health Practice Project (EPHPP) quality assessment tool was used to assess the methodological quality and risk of bias of all included studies. The tool was chosen as the included studies employed various study designs and EPHPP has been psychometrically validated for the evaluation of such studies (37, 38). Studies were rated as either ‘strong’, ‘moderate’, or weak’ across EPHPP’s six core subscales: *Selection Bias, Study Design, Confounders, Blinding, Data Collection Methods and Withdrawals and Drop-Outs.* For the ‘*Confounders’* subscale, studies that only sampled one group were rated ‘not applicable’ (N/A). As per EPHPP guidance, the six core subscale ratings were combined to provide each study with a global rating of ‘strong’, ‘moderate’, or weak’. Strong ratings were given to studies that did not have any weak component ratings. Moderate ratings were given to studies that had one weak component rating, and weak ratings were given to studies with two or more weak component ratings. In line with the design of EPHPP, each study was also evaluated on the *‘Intervention Integrity’ and ‘Analyses’* subscales but these did not influence global ratings.

An ‘ATT Fidelity and Adherence’ checklist was developed and used as a descriptive tool to assess the quality of ATT delivery (see **Supplementary Material - Table 1**). Fidelity referred to whether delivery was consistent with and grounded in the metacognitive theory, as suggested by Bellg and colleagues (39). Adherence referred to whether ATT was delivered in accordance with the treatment manual (2, 40). Ratings on the checklist informed item G2 (“was the consistency of the intervention measured?”) on the EPHPP ‘*Intervention Integrity’* subscale which was amended to “was ATT delivered as intended?”. All included studies were assessed for quality independently by the first author and a second reviewer which yielded fair interrater agreement (*κ* = 0.39). Identified reasons for initial disagreement were differences in interpretation of EPHPP criteria, namely the *Selection Bias, Confounder and Data Collection Methods* subscales and likely contamination of ratings by reporting standards. Each study was consequently reevaluated collaboratively which yielded substantial agreement (*κ* = 0.82). Discrepancies were discussed with the research team until agreement was reached.

### 2.6 Effect size estimation and suitability for sub-group meta-analyses

Where data was available, Cohen’s *d* was calculated for primary outcomes across all studies, except for the case study by Levaux and colleagues (41). However, this was not always possible where data was not reported, unavailable or where the primary measures of effect were not specified. Cohen’s *d* values of 0.8, 0.5 and 0.2 indicate large, medium and small effects, respectively (42). Most studies reported partial eta squared (*η_p_^2^*) (43) or eta-squared (*η^2)^*. *η_p_^2^* values of 0.14, 0.06, 0.01 indicate large, medium and small effects, respectively. Cohen’s 1969 (44) criteria cannot be applied to *η^2^*; therefore, direct comparison between studies must be interpreted with caution. There is also a possibility that studies reporting *η^2^* are a likely inflation of effect (45). A random effects model meta-analysis (46) was planned but was not conducted as there was a combination of insufficient numbers of studies using the same cognitive task and/or neural measure, significant heterogeneity between studies or insufficient data available.

## 3 Results

**Figure 1:**
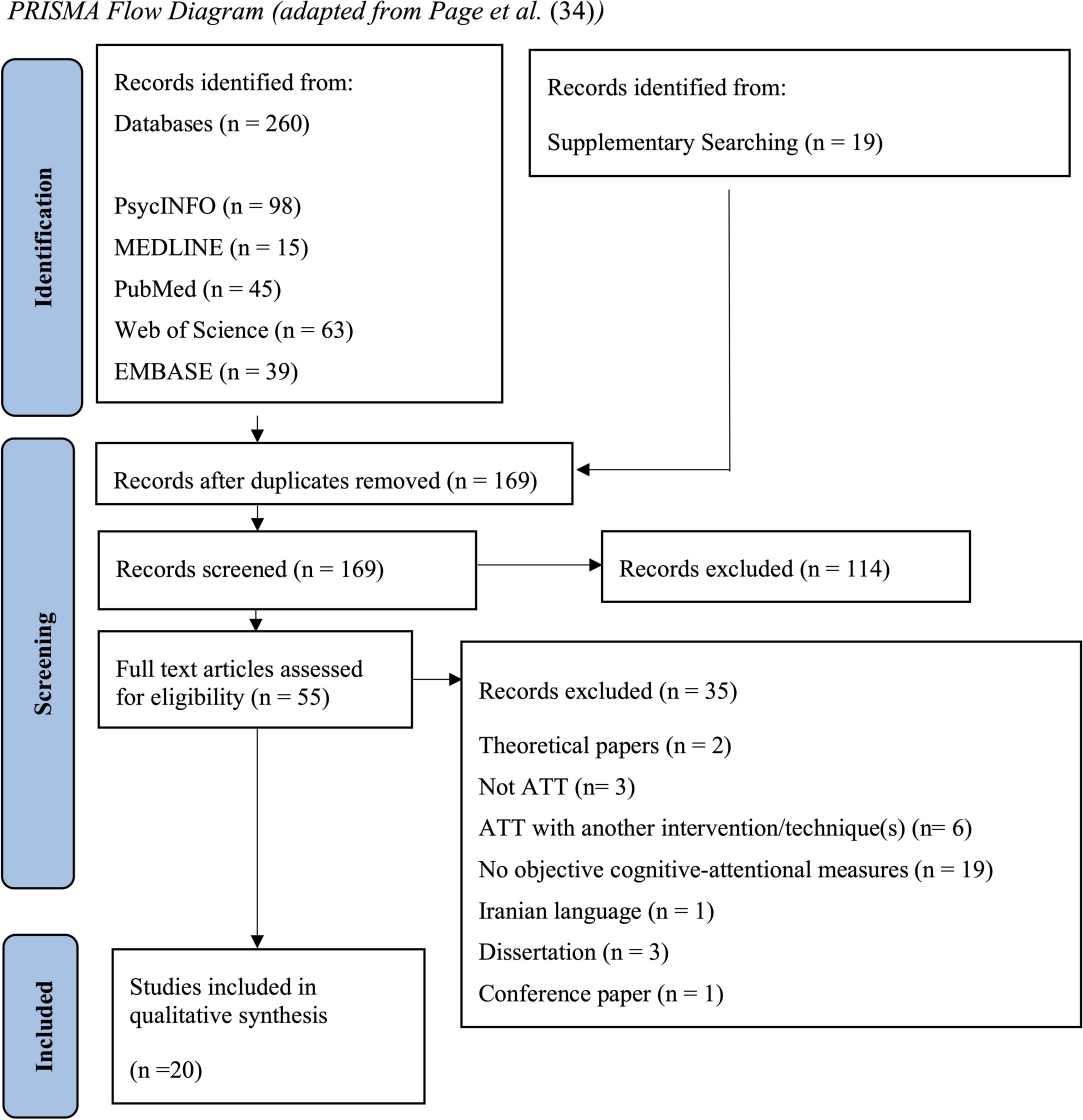
Flow diagram [adapted from Page et al. (34)].

The literature search yielded a total of 260 results. **Figure 1** outlines the screening process. After duplicates were removed, 169 records remained for screening. After screening the title and abstracts, 114 records were excluded. Of the 55 full texts screened, a total of 20 studies met full inclusion criteria.

### 3.1 Description of included studies

**Table 3** summarises the characteristics of each included study, conducted across eight different countries with over half (12/20) based in the UK or Germany. Most (17/20) studies used a randomised/controlled experimental design. Two studies used a pre-post design, and one was a case study. Overall, 1, 230 participants (62% female) were included with a mean age range of 5.87– 35.04 years. Three studies (19–21) reported data from an aggregated sample of two independent participant pools. All three studies were included; however, data was extracted separately from the two independent samples across all three studies to avoid duplication of participants. Other sociodemographic variables (e.g., ethnicity) were only reported in five studies, therefore were not used to describe the overall sample.

**Table 3:** Study characteristics.

Study	Country	Design	Population	Psychological morbidity	*N*	ATT – version	ATT – delivery	ATT -duration	ATT – dose	Home practice	Comparator(s)	Cognitive-attentional task(s)	Neural measure
Sharpe et al. (2010) (47)	Australia	Randomised experimental design	Adult	Pain*^NC^*	103	^T^ Wells (2008)	Audio	12 minutes	1	No	PMR	EDP	–
Levaux et al. (2011) (41)	Belgium	Case Study	Adult	Paranoid Schizophrenia	1	^T^ Adapted Wells (2000)	Therapist	15–30 minutes	9	Yes	Baseline	Text Comprehension; Story Learning; Word-List Learning + Cognitive Battery	–
Callinan et al. (2015) (16)	UK	Randomised experimental design	Adult	Traumatic Stress*^NC^*	60	^T^ Wells (2009)	Audio	12 minutes	2	Yes	Attention Filler Task	ACCE	–
Schwind et al. (2016) (48)	Germany	Controlled experimental design	Adult	Health Anxiety*^NC^*	54	^T^ Wells (1990)	Therapist	10 minutes	8	Yes	ATT_(body)_ No Intervention	EST	–
McEvoy et al. (2017) (24)	Australia	Randomised controlled design	Adult	Trait Anxiety*^NC^*	81	^T^ Wells (Unknown)	Audio	12 minutes	1	No	MB-PMR TWC	EST	–
Taraban et al. (2017) (49)	USA	Randomised experimental design	Adult	Mind-Wandering*^NC^*	43	^T^ Wells (2009)	Therapist	12 minutes	1	No	Unfocused Attention Induction	MWRA	–
Fergus & Hiraoka (2018) (50)	USA	Pre-Post	Adult	Anxiety disorder – *OCD; GAD; Panic; Social Anxiety; PTSD*	16	^T^ Wells (Unknown)	Therapist & Audio	12 minutes	13_Average_	Yes	Baseline	ANT	–
Barth et al. (2019) (19)	Germany	Randomised placebo-controlled experimental design	Adult	–	81	^E^ Wells (2009)	Audio - German	12 minutes	2-4	No	Sham	DL ANT EDP CWST 2-Back 3-Back	–
Fernie et al. (2019) (51)	UK	Randomised experimental design	Adult	High Anxiety and Worry*^NC^*	46	^T^ Wells (2019)	Audio	12 minutes	1	No	Sham	Modified CWST	–
Heitland et al. (2020) (20)	Germany	Randomised placebo-controlled experimental design	Adult	–	54	^E^ Wells (2009)	Audio - German	12 minutes	2, 4, 15	No	Sham	DL EDP CWST 2-Back	–
Stewart et al. (2021) (25)	Canada	Randomised experimental design	Adult	Probable GAD	78	^T^ Wells (2009)	Audio	12 minutes	7	Yes	Sham	ANT EDP	–
Murray et al. (2016)(23)	UK	Randomised experimental design	Child	–	100	^E^ Wells (1990)	Audio	11 minutes	3	No	No intervention	DNT	–
Murray et al. (2018) (22)	UK	Randomised experimental design	Child	–	101	^E^ Wells (1990)	Audio	11 minutes	3	No	PMR No intervention	DNT	
Knowles & Wells (2018)(28)	UK	Randomised controlled comparison	Adult	–	36	^E^ Wells (2009)	Audio	12 minutes	1	No	Passive ATT	–	EEG
Rosenbaum et al. (2018) (29)	Germany	Controlled experimental design	Adult	–	46	^E^ Wells (1990)	Audio	7x 40 second blocks	7	No	Passive ATT	d2 TOA	fNIRS
Kowalski et al. (2020) (30)	Poland	Randomised controlled comparison	Adult	–	89	^E^ Wells (2009)	Audio	12 minutes	1	No	Reverse ATT	d2 TOA CTT	fMRI
Usui et al. (2022) (52)	Japan	Pre-Post	Adult	–	20	^E^ Wells (2000)	Audio	9–15 minutes	1 (+14-20)	No	Baseline	–	EEG
Jahn et al. (2023) (21)	Germany	Randomised experimental design	Adult	–	51	^E^ Wells (2009)	Audio - German	12 minutes	16	Yes	Sham	EDP CWST DL 2-back	fMRI
Müller et al. (2025) (53)	Germany	Randomised experimental design	Adult	MDD	99	^E^ Wells (2009)	Audio - German	12-minutes	16	Yes	Sham	–	fMRI
Schwarz et al. (2025) (54)	Germany	Controlled experimental design	Adult	–	71	^E^ Wells (2009)	Audio - German	Sample 1: 4x 35 second blocks Sample 2: 4x 20 second blocks	1	No	Passive ATT (CON_(ATT)_) Alternative sounds (CON_(sounds)_) White noise (CON_(white)_)	–	fMRI

^NC^, Non-clinical population; GAD, Generalised Anxiety Disorder; MDD, Major Depressive Disorder; ^T^, Treatment; ^E^, Experimental; PMR, Progressive muscle relaxation; MB-PMR, Mindfulness-based progressive muscle relaxation; ATT_(body),_ Adaptation of ATT directing attention towards bodily sensations; TWC, Thought-wandering control; EDP, Emotional dot-probe; ACCE, Attentional Control Capacity for Emotional Representations; EST, Emotional Stroop task; MWRA, Mind-wandering Reading Algorithm; ANT, Attention Network Task; DL, Dichotic Listening task; CWST, Colour-Word Stroop task; DNT, Day/Night Task; d2 TOA, d2 Test of Attention; CTT, Colour-Trail Test; ^NR^, Not reported; EEG, Electroencephalography; fNIRS, Functional Near-infrared Spectroscopy; fMRI, functional Magnetic Resonance Imaging; CON, Control.

Four studies used a clinical sample (25, 41, 50, 53). Two of which (25, 50) recruited participants who had either received a clinical diagnosis or met diagnostic criteria for an Anxiety Disorder or Post-Traumatic Stress Disorder (PTSD) according to the Diagnostic and Statistical Manual of Mental Disorders (5^th^ ed.; DSM-V) (55); one study (41) recruited an individual with a diagnosis of paranoid schizophrenia according to the DSM-IV (56); and the fourth (53) recruited individuals who had a diagnosis of Major Depressive Disorder (MDD) according to the DSM-IV (56). Four studies (16, 24, 48, 51) recruited participants without a clinical diagnosis but with self-reported heightened levels of anxiety or recent experience of a stressful life event. Two studies (47, 49) used experimental paradigms to induce the presenting problem of interest in healthy participants (e.g., pain and mind-wandering), hereafter referred to as non-clinical samples. Ten studies (19–23, 28–30, 52, 54) used healthy samples, two of which were with school-aged children.

Methodologies across studies were mixed. In the 17 studies that utilised a randomised/controlled experimental design, there were different comparators. Four studies (22, 24, 47, 48) used an active treatment comparator. Schwind and colleagues (48) created an adapted version of ATT (ATT_(body)_), that trained attention inwards towards bodily sensations. The other three studies used a Progressive Muscle Relaxation (PMR) exercise. Additionally, three of the four studies had more than one comparator condition. McEvoy and colleagues (24) also used an active control comparator. Schwind and colleagues (48) and Murray and colleagues (22) also used a no intervention condition. Six other studies (19–21, 25, 51, 53) used only sham ATT. Five studies (16, 28–30, 49) used only an active control comparator and one (23) used only a no intervention condition. In an attempt to isolate the active component of ATT, Schwarz and colleagues (54) carefully selected three distinct control conditions that varied in audio-input complexity.

There were inconsistencies in reporting of key methodological factors including attrition, follow up, and power analyses. Attrition was only reported in one study (*n* = 4) (25) and only one study (41) conducted follow up. Ensuring a study has sufficient power is imperative, however power analyses were only completed in seven studies (36.8%) (21, 22, 24, 25, 47, 48, 51). Six studies (16, 19, 30, 49, 53, 54) discussed power but did not report completing a power analysis. While 17 studies included effect sizes, these were inconsistently reported.

### 3.2 Quality assessment

Global EPHPP ratings are outlined in **Table 4** (see **Supplementary Material - Table 2** for individual EPHPP ratings). Two studies (22, 23) received ‘strong’ ratings; eleven studies (16, 19, 21, 28–30, 47, 48, 51, 53, 54) received ‘moderate’ ratings and seven studies (20, 24, 25, 41, 49, 50, 52) received ‘weak’ ratings. Moderate and weak ratings were predominantly the result of weak scores on the ‘*Selection Bias’* and/or ‘*Blinding’* subscales. Except for participants from two studies by Murray and colleagues (22, 23), study samples were considered unlikely to be representative of the target population, because of self-referral recruitment processes or less than 60% agreement to participate after screening. Although most studies (11/20) used at least single-blinding, eight studies either did not clearly report whether blinding was used or it was inappropriate to the study design. Ratings across the ‘*Study Design’, ‘Confounders’, ‘Data Collection Methods’* and *‘Withdrawals and Dropout’* subscales were areas of strength for each study. Studies that scored lower on these subscales were predominantly due to study design or unclear reporting. Statistical analyses were considered appropriate for the research questions across all studies.

**Table 4:** Quality appraisal and intervention integrity of included studies.

Study	Selection bias	Study design	Confounders	Blinding	Data Collection methods	Withdrawals and dropouts	Intervention integrity	Overall rating
Sharpe et al. (2010) (47)	Weak	Strong	Strong	Moderate	Strong	Strong	Audio rationale omitted, written provided. No report of metacognitive-level dialogue. Advised as a coping strategy.	Moderate
Levaux et al. (2011) (41)	Weak	Weak	N/A	Weak	Weak	Moderate	Each component administered separately.	Weak
Callinan et al. (2015) (16)	Weak	Strong	Strong	Moderate	Strong	Strong	No report of metacognitive-level dialogue.	Moderate
Schwind et al. (2016) (48)	Weak	Strong	Strong	Moderate	Strong	Strong	Omitted rationale and no report of credibility check, SAR or metacognitive-level dialogue.	Moderate
McEvoy et al. (2017) (24)	Weak	Strong	Weak	Moderate	Strong	Strong	No report of credibility check, metacognitive-level dialogue or home-practice.	Weak
Taraban et al. (2017) (49)	Weak	Strong	Strong	Weak	Strong	Strong	Yes	Weak
Fergus & Hiraoka (2018) (50)	Weak	Moderate	N/A	Weak	Strong	Strong	Replaced rationale with ACT metaphor (Harris, 2009).	Weak
Barth et al. (2019) (19)	Weak	Strong	Strong	Moderate	Strong	Strong	No report of SAR.	Moderate
Fernie et al. (2019) (51)	Weak	Strong	Strong	Moderate	Strong	Strong	No report of metacognitive-level dialogue.	Moderate
Heitland et al. (2020) (20)	Weak	Strong	Weak	Moderate	Strong	Strong	No report of metacognitive-level dialogue.	Weak
Stewart et al. (2021) (25)	Weak	Strong	Weak	Moderate	Strong	Strong	No report of metacognitive-level dialogue.	Weak
Murray et al. (2016) (23)	Moderate	Strong	Strong	Moderate	Strong	Strong	No report of credibility check, SAR or metacognitive-level dialogue.	Strong
Murray et al. (2018) (22)	Moderate	Strong	Strong	Moderate	Strong	Strong	No report of credibility check, SAR or metacognitive-level dialogue.	Strong
Knowles & Wells (2018) (28)	Weak	Strong	Strong	Moderate	Strong	Strong	No report of metacognitive-level dialogue.	Moderate
Rosenbaum et al. (2018) (29)	Weak	Strong	Strong	Moderate	Strong	Moderate	N/A dismantled into experimental paradigm.	Moderate
Kowalski et al. (2020) (30)	Weak	Strong	Strong	Strong	Strong	Strong	No report of metacognitive-level dialogue.	Moderate
Usui et al. (2022) (52)	Weak	Moderate	N/A	Moderate	Strong	Weak	Yes	Weak
Jahn et al. (2023) (21)	Weak	Strong	Strong	Strong	Strong	Strong	No report of credibility check, SAR or metacognitive-level dialogue.	Moderate
Müller et al. (2025) (53)	Weak	Strong	Strong	Strong	Strong	Strong	Reported to administer in line with Wells (2009) but does not report use of credibility check, SAR or metacognitive-level dialogue.	Moderate
Schwarz et al. (2025) (54)	Weak	Strong	Strong	Moderate	Strong	Strong	N/A dismantled into experimental paradigm.	Moderate

Green, orange and red colour shading indicates studies that received a 'strong', 'moderate' and 'weak' methodological quality rating, respectively.

### 3.3 ATT fidelity

Nine studies evaluated ATT as an experimental treatment for clinical or non-clinical samples; and eleven studies used ATT experimentally to explore its mechanisms, two of which (29, 54) dismantled the ATT procedure into its component parts. All studies were grounded in Wells’ metacognitive theory (1, 2). ATT was delivered as an audio-recording in 17 studies; 16 of which cited the source from where the recording was obtained. Five studies (19, 20, 53, 54) used German versions of audio-recorded ATT. Three studies used therapist-guided delivery (41, 48, 49) and reported following the 2009 script by Wells (2). Fergus and Hiraoka (50) used therapist-guided delivery plus audio-recordings for home practice. Eleven studies reported permission to use ATT. Only four studies (22, 23, 28, 29) reported that training was received on how to administer ATT. The version of ATT used was not always explicitly reported.

### 3.4 ATT adherence

Given the difference between studies exploring ATT as an experimental treatment versus understanding its mechanisms by dismantling the technique, the assessment of adherence was broadened to whether studies used all of the individual components of ATT as per protocol. When used as a treatment package, all three components of ATT should be practiced in a “*single seamless exercise”* comprised of multiple sounds at varying spatial locations to ensure it is sufficiently attentionally demanding (p.57, 2). Out of the eighteen studies intending to utilise the ATT as a single exercise lasting between 9 and 14 minutes, one (41) taught the ATT components separately across nine 15 to 30 minute sessions to control for learning pace. The two dismantling studies (29, 54) presented each component of interest individually and repeatedly within specified experimental blocks, as intended. Seventeen studies described ATT to include multiple sounds at varying spatial locations; however, this was not mentioned in three (29, 49, 52).

The full ATT was inconsistently used, including in those using it experimentally. In usual clinical practise it should be introduced using a credible and acceptable rationale. Eighteen studies used a rationale based in metacognitive theory. However, one study (48) omitted the rationale and another (50) used a theoretically incompatible metaphor from a different therapeutic model (57). Note the two dismantling studies have been excluded from the assessment here. In clinical practise, use of a credibility check (58) is recommended to determine the extent of perceived therapeutic benefit. Only one study (25) reported completing a credibility check. Of particular importance in clinical use, a Self-Attention Rating (SAR) (2) is used as a guide to assess the efficacy of ATT in counteracting the CAS, in which a two-point shift from internal to externally focused attention is a minimal mechanistic marker of successful implementation. If this is not achieved in the first session then the procedure should be repeated. Six studies (16, 24, 25, 30, 47, 54) used a SAR rating; however, only two of these studies (16, 25) made it clear that the two-point criterion was applied. Eliciting feedback through a metacognitive dialogue that explores and shapes a person’s experience is an integral part of ATT when used in treatment to facilitate the necessary shift towards a metacognitive mode of processing and in identifying and resolving any ongoing CAS-related activity during practice. Three studies (41, 50, 52) reported using exploration and shaping of patient experience; however, this was unclear in the majority (*n* = 15). Homework practice is a usual component of ATT when used as a therapy. Although six studies (24, 28, 30, 47, 49, 51) specifically aimed to experimentally evaluate a single practice of ATT, nine (16, 20, 21, 25, 41, 48, 50, 52, 53) out of an expected 12 studies used home practice. Home practice varied between twice per week to once or twice daily for between six and 28 days. ATT guidelines explicitly state that it is not to be used as a coping strategy in response to difficult thoughts and feelings, to prevent it from becoming a form of avoidance that keeps individuals locked within the CAS. One study (47) incorrectly advised ATT to be used as a coping strategy during an in-study task.

## 4 Findings across cognitive attentional tasks and neural measures

### 4.1 Description of cognitive-attentional tasks

**Table 5** summarises the results by task. Fourteen cognitive-attentional tasks were used across 16 studies. The number of tasks used within each study ranged between one and five. Six studies used versions of the Stroop task (59): four used a colour-word Stroop (59) and two used versions of the emotional Stroop (60). Two studies used the Day-Night task (61). Five studies used the emotional dot probe (62). One study used a modified version of the Attentional Control Capacity for Emotional Representations task (63, 64). Three studies used versions of the Attention Network Task (65, 66). Three studies used the Dichotic Listening task (67). Three studies used the d2 Test of Attention (68, 69). One study used a Polish adaptation of the Colour-Trail Test (70). Three studies used versions of the N-Back test (71). One study used a Mind Wandering Reading Algorithm (72). The case study by Levaux and colleagues (41) used three objective ‘ecological’ measures and completed a neuropsychological battery comprised of ten subtests. Across each group of tasks there was substantial variability in the number of trials and approaches taken to task scoring/analysis. See **Supplementary Material - Table 3** for task descriptions.

**Table 5:** Cognitive-attentional task performance results (effect sizes) by study.

Study	Sample	Task	*d*	*η_p_^2^*	*η^2^*	*r_s (ACS)_*	*p*
*Fernie* et al. *(2019) (*51*)*	Adult-NC	CWST	RT: 0.84 GI: 0.84	-	-	-	RT: 0.009* GI: 0.016*
			PL RT: 1.11 PL GI: 1.01				RT: 0.001* GI: 0.001*
*Barth* et al. *(2019) (*19*)*	Adult-H		~0.20	0.04	-	-	0.081
*Heitland* et al. *(2020) (*20*)*	Adult-H		NR	NR	–	-0.48	>0.102 0.009*
*Jahn* et al. *(2023) (*21*)*	Adult-H		~0.04	0.002	-	-	>0.065
*Schwind* et al. *(2016) (*48*)*	Adult-NC	EST	Illness: ~0.37 Body: ~0.27 Panic: ~0.23	Illness: 0.12 Body: 0.07 Panic: 0.05	-	–	Illness: 0.04* Body: 0.18 Panic: 0.26
*McEvoy* et al. *(2017) (*24*)*	Adult-NC		-	-	Threat Speed: 0.000 Neutral Speed: 0.004 Threat Errors: 0.025* Neutral Errors: 0.001	-	Threat Speed: 0.99 Neutral Speed: 0.59 Threat Errors: 0.025* Neutral Errors: 0.85
*Murray* et al. *(2016) (*23*)*	Child-H	DNT	~0.02	<0.001	-	-	0.960
*Murray* et al. *(2018) (*22*)*	Child-H		~0.31	0.09	-	-	0.01*
*Sharpe* et al. *(2010) (*47*)*	Adult-NC	EDP	H-Sensory: ~0.23 H-Affective: ~0.18 Disengage: NR	H-Sensory: 0.05 H-Affective: 0.03 Disengage: NR	-	-	H-Sensory: 0.033* H-Affective: 0.093 Disengage: NR
*Barth* et al. *(2019) (*19*)*	Adult-H		NR 4^ATT^Emot: ~0.27 4^ATT^Neut: ~0.34 4^ATT^Corres: ~0.26	NR 4^ATT^Emot: 0.071 4^ATT^Neut: 0.106 4^ATT^Corres: 0.066	-	-	0.89 4^ATT^Emot-Neut: 0.59 4^ATT^Emot: 0.08 4^ATT^Neut: 0.031* 4^ATT^Corres: 0.092
*Heitland* et al. *(2020) (*20*)*	Adult-H		~0.43	0.158	–	4^ATT^ = -0.451	0.006* 0.027*
*Stewart* et al. *(2021) (*25*)*	Adult-C		NR	-	-	-	NR
*Jahn* et al. *(2023) (*21*)*	Adult-H		Bias: ~0.30 Orienting: ~0.07 Disengage: ~0.38	Bias: 0.084 Orienting: 0.005 Disengage: 0.129	–	–	Bias: 0.05 Orienting: 0.63 Disengage: 0.015
*Callinan* et al. *(2015) (*16*)*	Adult-NC	ACCE	Happy: ~0.37 Angry: ~0.35 Neutral: ~0.10	Happy: 0.12 Angry: 0.11 Neutral: 0.01	-	-	Happy: 0.008** Angry: 0.012* Neutral: 0.559
*Fergus and Hiraoka (2018) (* 50 *)*	Adult-C	ANT	Alert: 0.49 Orient: 0.15 EA: 0.9	–	–	–	Alert: 0.147 Orient: 0.622 EA: 0.005*
*Barth* et al. *(2019) (*19*)*	Adult-H		UTC	-	-	-	Alert: 0.28 Orient: 0.53 EA: 0.92
*Stewart* et al. *(2021) (*25*)*	Adult-C		NR	–	–	–	NR
*Barth* et al. *(2019) (*19*)*	Adult-H	DL	~0.26	0.065*	-	-	0.026
*Heitland* et al. *(2020) (*20*)*	Adult-H		~0.31	0.085*	–	–	0.047
*Jahn* et al. *(2023) (*21*)*	Adult-H		~0.25	0.06	-	-	0.086
*Rosenbaum* et al. *(2018) (*29*)*	Adult-H	d2 ToA	Screening Only				
*Kowalski* et al. *(2020) (*30*)*	Adult-H		-	-	TOT: 0 %Errors: 0.01 CP: 0	-	TOT: 0.95 %Errors: 0.42 CP: 0.61
*Levaux* et al. *(2011) (*41*)*	Adult-C		–	–	–	–	NR – Raw Scores Only
*Kowalski* et al. *(2020) (*30*)*	Adult-H	CTT			CTT1: 0 CTT2: 0	-	CTT1: 0.33 CTT2: 0.48
*Barth* et al. *(2019) (*19*)*	Adult-H	3-Back	NR	NR	-	-	0.55
*Barth* et al. *(2019) (*19*)*	Adult-H	2-Back	NR	NR	-	-	0.77
*Heitland* et al. *(2020) (*20*)*	Adult-H		NR	NR	–	-0.684	0.457 <0.001*
*Jahn* et al. *(2023) (*21*)*	Adult-H		Target counts: ~0.11 Target RTs: ~0.11	Target counts: 0.012 Target RTs: 0.012	-	-	Target counts: 0.436 Target RTs: 0.435
*Taraban* et al. *(2017) (*49*)*	Adult-NC	MWRA	~0.6	-	-	-	0.03*
*Levaux* et al. *(2011) (*41*)*	Adult-C	Text Comprehension	-	-	-	-	NR
		Story Learning	–	–	–	–	Immediate: < 0.001 Delayed: < 0.001
		Word-List Learning	-	-	-	-	< 0.001

*p < 0.05. H, Healthy; NC, Non-Clinical; C, Clinical; CWST, Colour-Word Stroop; EST, Emotional Stroop; DNT, Day Night Task; EDP, Emotional Dot Probe; ACCE, Attentional Control Capacity for Emotional Representations; ANT, Attention Network Task; DL, Dichotic Listening; d2 ToA, d2 Test of Attention; CTT(1/2), Colour Trail Test (Versions 1 and 2); MWRA, Mind Wandering Reading Algorithm; RT, Reaction Time; GI, Global Index; PL, Perceptual Load; NR, Not Reported; Alert, Alerting Index; Orient, Orienting Index; EA, Executive Attention Index; UTC, Unable to calculate with available data; TOT, Total Number of Characters Processed; %Errors, Percentage of Errors; CP, Concentration Performance.

#### 4.1.1 Stroop

##### 4.1.1.1 Colour-word stroop

ATT-dependent CWST performance varied across non-clinical and healthy samples. One of four studies (51) found significantly improved CWST performance after one ATT dose in a high worry/anxiety sample with large effect sizes (Global Index (GI) *d =* 0.84; Reaction Time (RT) *d =* 0.84) in comparison to sham ATT. Whilst all participants significantly improved across time-points, the effect size and rate of improvement was consistently larger post-ATT, particularly under conditions of high perceptual load (GI *d* = 1.11; RT *d* = 1.01). In contrast, three studies (19–21) evaluating two, four and fifteen doses of ATT with healthy samples found no significant ATT-dependent (or dose dependent) CWST effects. However, Barth and colleagues (19) found a non-significant trend for faster incongruent minus congruent RTs following ATT in comparison to a sham control condition (*η_p_^2^* = 0.04). Whilst Heitland and colleagues (20) found no associations between baseline scores on the Attentional Control Scale (ACS) (73) and CWST performance measured after two, fifteen or sham doses of ATT; they found those with a higher ACS score at baseline showed significantly larger CWST improvements after four doses of ATT. As such, there are inconsistent and mixed results for ATT-dependent CWST effects, depending on target population, number of doses and perceived level of attentional control at baseline. Such variability may be attributable to insufficient numbers of trials and statistical power; however, differences might exist between the original English and German translation/version of the ATT audio-recording across these studies. However, perceived attentional control at baseline might influence the effect of ATT on CWST performance making any relationship more complex.

##### 4.1.1.2 Emotional stroop

There were no significant ATT-dependent EST effects found across two studies using non-clinical samples. However, McEvoy and colleagues (24) found a significant interaction for threat errors in an elevated trait anxiety sample; mean errors for threatening words decreased after a single ATT session (*d* = 0.36) and mindfulness-based PMR practice (*d* = 0.50) but increased in the control group (*d* = -0.44). However, these between group differences failed to reach statistical significance. Similarly, Schwind and colleagues (48) found no ATT-dependent effects in a sample with elevated health anxiety after eight ATT doses. They found that ATT_body_ yielded significant reductions in attentional bias towards illness words (*d* = 0.93), but not ATT (*d =* 0.06) nor the control group (*d =* 0.33). No significant effects were found for bodily complaints or panic words. Interpretation of the lack of significant EST findings is compounded by the absence of a metacognitive rationale in Schwind et al. (48) and the absence of meta-level dialogue in both studies. Both studies also failed to show the desired shift towards externality normally used as indicating correct ATT implementation.

#### 4.1.2 Day-night task

Two studies used the DNT with classrooms of primary school children, and one study found an effect. Murray and colleagues (23) found no significant ATT-dependent effects on verbal DNT performance (*η_p_^2^* ≤ 0.001). Whereas Murray and colleagues (22) found large significant effects (*η_p_^2^* = .09), with the rate of improvement post-ATT significantly differing from the no intervention control group but not the relaxation (PMR) group. The difference between these two study findings may have been influenced by high baseline DNT performance in Murray et al. (23).

The two DNT studies stand out among the other papers in the review as they involve young children and the primary measure in the study was delay of gratification measured behaviourally with the Marshmallow task. In both studies, ATT was found to increase the ability to delay eating a treat. Such delay is considered to be an index of executive control in the domain of response inhibition.

#### 4.1.3 Emotional dot probe

Unlike inconsistent effects across Stroop studies, four of five studies found significant ATT-dependent EDP effects. Three studies (19–21) using healthy samples showed that two, four and fifteen ATT doses facilitated faster attentional disengagement from irrelevant and/or emotional stimuli and towards relevant stimuli, with a potential dose effect. Barth and colleagues (19) found participants receiving four ATT doses responded significantly faster to neutral stimuli (*η_p_^2^* = 0.106) and demonstrated a non-significant trend for faster RTs to both emotional and corresponding stimuli in comparison to the sham group (*η_p_^2^* = 0.071, 0.066, respectively). Heitland and colleagues (20) also found those receiving 15 ATT doses showed faster attentional disengagement (*η_p_^2^* = 0.158) compared to sham. For those who received four ATT doses, a high ACS score at baseline was associated with improved disengagement abilities (*r_s_*= -0.451). Jahn and colleagues (21) found ATT yielded an improvement in responding to incongruent stimuli (*η_p_^2^* = 0.169) but not congruent (*η_p_^2^* = 0.07) or neutral (*η_p_^2^* = 0.031) stimuli. ATT yielded significantly faster attentional disengagement (*η_p_^2^* = 0.174), even after controlling for covariates of gender and age (*η_p_^2^* = 0.129). A non-significant trend was also found post-ATT for the bias index (*η_p_^2^* = 0.084), but no effect was found for the orienting index (*η_p_^2^* = 0.005). Region of Interest (ROI) functional Magnetic Resonance Imaging (fMRI) analyses found those receiving ATT demonstrated decreased activation in the ACC when presented with incongruent stimuli (*p* = 0.045), however no ATT-dependent effects were shown.

These findings contrast to those of Sharpe and colleagues (47) who found significant effects associated with ATT in a sample with induced pain on hypervigilance but not attentional disengagement. Those receiving ATT became significantly less hypervigilant towards sensory pain words, whilst those receiving PMR changed in the opposite direction (*η_p_^2^* = 0.05). There were no significant effects found for hypervigilance to affective pain words (*η_p_^2^* = 0.03) or attentional disengagement from pain-related stimuli.

One study (25) found no ATT-dependent EDP effects in a sample with probable Generalised Anxiety Disorder (GAD). Those receiving ATT demonstrated greater difficulty disengaging from threat compared to the sham control group (*η_p_^2^* = 0.07) but this did not change over time. These findings may be accounted for by participants reporting more internalised attention by the end of the intervention which is at odds with the goal of ATT. Such effects may reflect the quality of the procedure when used clinically in the absence of a metacognitive-level dialogue. Overall, ATT-dependent EDP effects were found; ATT was associated with enhanced attentional disengagement in healthy and non-clinical samples, however greater inconsistencies were present in clinical samples.

#### 4.1.4 Attentional control capacity for emotional representations

ATT-dependent ACCE performance effects were found in one study which suggested that ATT modifies processing strategy dependent on task demands in participants reporting distress because of stressful life events (16). One ATT dose resulted in significantly slower disengagement from happy faces and faster disengagement from angry faces in comparison to the control group. After the second ATT dose, the ATT group disengaged from happy faces significantly faster than the control group (*η_p_^2^* = 0.12) whereas the control group disengaged significantly faster than the ATT group from angry faces (*η_p_^2^* = 0.11). There was no significant difference between groups for neutral faces (*η_p_^2^* = 0.01). These findings suggest a change in the prioritisation of threat-related stimuli following ATT but must be held tentatively as a true baseline was not established.

#### 4.1.5 Attention network task

Variation in ATT-dependent ANT performance effects were found across clinical and healthy samples. Fergus & Hiraoka (50) found large and significant improvements in the executive control ANT index in a clinically anxious sample following an average of 13 ATT doses (*d* = 0.9). In contrast, there were no significant differences found in the alerting (*d* = 0.49) or orienting ANT indices (*d =* 0.15). Two other studies did not find ATT-dependent effects on any ANT index in healthy (19) or clinical samples (GAD) (25) after two, four and seven ATT doses, respectively.

#### 4.1.6 Dichotic listening task

Two of three studies (19, 20) found significant improvements in auditory selective attention in healthy samples after two and four (*η_p_^2^* = 0.065) and 15 ATT doses (*η_p_^2^* = 0.085). In contrast, Jahn and colleagues (21) found no significant differences between healthy ATT and control groups but a non-significantly greater reduction in RTs after 15 ATT doses compared to sham. Whilst single-ear analysis (of both left and right ears) showed a non-significant difference between ATT and the sham ATT, left ear RTs did show a non-significant trend towards greater improvement post-ATT (*η_p_^2^* = 0.06). Individual differences in asymmetries were considered a potential reason that may account for discrepancies in findings. There was no association found between ACS score at baseline and selective auditory attention improvements after two, four or fifteen ATT doses (20).

#### 4.1.7 d2 test of attention

Although three studies used the d2 TOA, Rosenbaum and colleagues (29) used it as a screening tool only and Levaux and colleagues (41) used it within a cognitive battery reporting only raw and standardised scores with no further details provided on test administration or scoring. Kowalski and colleagues (30) found no significant ATT-dependent effects for working attention speed, accuracy, or concentration performance (all *η^2^* = 0) in participants presenting with high and low levels of CAS. On the other hand, Levaux and colleagues (41) found that one participant with a diagnosis of Schizophrenia demonstrated raw and standardised score improvements in all domains except for processing speed post-ATT, which were maintained at follow-up.

#### 4.1.8 Colour-trail test

Kowalski and colleagues (30) found no significant ATT-dependent effects on CTT performance in either the CTT1 or CTT2 (*η^2^* = 0) in individuals with high and low levels of CAS. The focus of attention appeared to be internalised following ATT which may account for these findings, suggesting ATT did not work as intended in this sample.

#### 4.1.9 N-back

No significant differences were found between ATT and sham for either the 3-back or 2-back tasks following two, four, or fifteen ATT doses across three studies using healthy samples (19 – 21). A higher ACS score at baseline was associated with larger ATT-dependent performance improvements in the 2-back task after four ATT doses, but not two or fifteen (20).

#### 4.1.10 Mind wandering reading algorithm

Taraban and colleagues (49) found significant between-group differences in the frequency of mind wandering (*p* = 0.03) across healthy participants. Those who received ATT demonstrated 60% less mind wandering than the control group.

#### 4.1.11 Text comprehension; story learning; word list learning

Of three ecological tasks used in the case study by Levaux and colleagues (41), significant improvements were found on two. The participant with a diagnosis of Schizophrenia showed no significant changes on the recognition component of a ‘Text Comprehension’ task, but showed significant improvements on the ‘Story Learning’ and ‘Word-List Learning’ tasks post-ATT.

#### 4.1.12 Cognitive battery

Levaux and colleagues (41) also administered ten neuropsychological subtests pre- and post-ATT. These included Forward and Backward Digit Span (74), Alpha Span (75), Test for Attentional Performance (TAP) (76); Go/no-go TAP; Incompatibility TAP; Hayling (77), Errand Test (78), aforementioned d2 ToA (79), Rappel Libre/Rappel Indicé-16 (80). Post-ATT raw score improvements were found in selective attention, distraction resistance, and flexibility, in addition to verbal episodic memory and working memory, all of which were maintained at follow-up.

### 4.2 Description of neural measures

Three neural measures; electroencephalography (EEG), functional near-infrared spectroscopy (fNIRS), functional magnetic resonance imaging (fMRI), were used across six studies using healthy samples (21, 28–30, 52, 54) and one study using a clinically depressed sample (53) (**Table 6**).

**Table 6:** Results by neural measure.

Study	Measure	Analysis							*d*				*r*	
				Condition	Frequency	ROI								
Knowles and Wells (2018) (28)	EEG	Spectral Analysis	Tonic Power Change	EO	Alpha	Anterior	0.487						–	
						Midline	0.800*						–	
						Posterior	0.130						–	
					Beta	Anterior	0.567						–	
						Midline	0.529						–	
						Posterior	0.114						–	
					Theta	Anterior	0.330						–	
						Midline	0.062						–	
						Posterior	0.238						–	
				EC	Alpha	Anterior	0.763*						–	
						Midline	0.457						–	
						Posterior	-0.421						–	
					Beta	Anterior	0.479						–	
						Midline	0.229						–	
						Posterior	-0.214						–	
					Theta	Anterior	0.453						–	
						Midline	-0.206						–	
						Posterior	-0.222						–	
			Band Power Change Associations	EO	Alpha-Beta	Anterior	~2.97						-0.83	
						Midline	~1.15						-0.50	
				EC	Alpha-Beta	Anterior	~1.42						0.58	
						Midline	~2.41						0.77	
			Within group correlations between ROIs	EO	Alpha	Ant-Mid	~1.85						0.68	
					Beta	Ant-Mid	~1.96						0.7	
				EC	Alpha	Ant-Mid	~1.76						0.66	
					Beta	Ant-Mid	~2.66						0.8	
			Alpha Asymmetry										-0.92	
				Condition	Frequency		DA			IR			DA	IR
Usui et al. (2022) (52)		sLORETA Imaging		Pre-Post Divided Attention (DA) and Initial Resting (IR)	Alpha-1 (-15,35,-25)		~0.45			~0.43			0.22	0.21
					Alpha-1 (-20,35,-25)		~0.34			~0.40			0.17	0.20
					Alpha-1 (-10,30,-20)		~0.40			~0.38			0.20	0.19
					Alpha-1 (-25,40,-20)		~0.40			~0.65			0.20	0.31
					Alpha-1 (-10,30,-15)		~0.35			~0.26			0.18	0.13
					Alpha-1 (-20,40,-20)		~0.18			~0.53			0.09	0.26
					Alpha-1 (-10,25,-15)		~0.32			~0.43			0.16	0.21
					Alpha-1 (-5,25,-10)		~0.36			~0.38			0.18	0.19
					Alpha-2 (50,-40,-15)		~1.09			~0.06			0.48*	0.03
Study	Measure	Analysis					*d*						*r*	
				Condition	Frequency		DA			IR			DA	IR
Usui et al. (2022) (52)	EEG	sLORETA Imaging			Alpha-2 (50,-40,-20)		~1.00			~0.28			0.45*	0.14
					Alpha-2 (55,-40,-15)		~1.09			~0.16			0.48*	0.08
Study	Measure	Analysis		ROI	Condition		η^2^						d	
Rosenbaum et al. (2018) (29)	fNIRS	Optical Imaging		R IFG	–		0.087**						–	
				R dlPFC	–		0.060*						–	
				SAC	–		0.078*						–	
				L IFG	–		0.050						–	
				L dlPFC	–		0.053						–	
				R IFG	Selective		–						0.33*	
					Switching		–						0.50**	
					Divided		–						0.33*	
				SAC	Selective		–						0.32*	
					Switching		–						0.45**	
					Divided		–						0.43*	
Study	Measure	Analysis					η^2^						d	
Kowalski et al. (2020) (30)	fMRI	Seed-to-Voxel Functional Connectivity		Seed	Voxel									
				Rumination-Induction Procedure (RUM):										
				FPN: L Posterior Parietal Cortex (-46 -58 49)	R-middle frontal gyrus (46 18 30)		0.08*						–	
				FPN: R Lateral Prefrontal Cortex (41 38 30)	L-inferior temporal gyrus (-62 -50 -16)		0.00						–	
					R-middle temporal gyrus (56-52-2)		0.01						–	
				FPN: R Posterior Parietal Cortex (52 -52 45)	R-middle temporal gyrus (56-48-2)		0.02						–	
				Abstract Thinking Procedure (ATP):										
				DMN: Precuneus Cortex (1 -61 38)	L-lateral occipital cortex (-30-78 10)		0.01						–	
					R-lateral occipital cortex (34-80 16)		0.00						–	
				Resting State								
				DAN: Intraparietal sulcus (39-42 54)	Bilateral precuneus cortex (-2-60 60)		0.06*						–	
Study	Measure	Analysis					η^2^						d	
Kowalski et al. (2020) (30)	fMRI	Inter-Subject Correlation		Neural Network	ROI		M_CAS		M_ATT		Interact			
				DAN	L-frontal eye field		0.30**		0.02		0.01		–	
					L-intraparietal sulcus		0.00		0.42***		0.05***		–	
					R-frontal eye field		0.00		0.28***		0.01		–	
					R-intraparietal sulcus		0.00		0.52***		0.01		–	
				DMN	L-lateral parietal cortex		0.03		0.45***		0.00		–	
					R-lateral parietal cortex		0.00		0.33***		0.00		–	
					Medial prefrontal cortex		0.02		0.36***		0.00		–	
					Precuneus cortex		0.05***		0.41***		0.03*		–	
				FPN	L-lateral prefrontal cortex		0.09***		0.46***		0.07***		–	
					L-posterior parietal cortex		0.01		0.48***		0.01		–	
					R-lateral prefrontal cortex		0.00		0.24***		0.00		–	
					R-posterior parietal cortex		0.01		0.22***		0.00		–	
Jahn et al. (2023) (21)		ROI Analysis		ACC									~0.6	
Müller et al. (2025) (53)		Seed-to-Voxel Functional Connectivity		Seed	Voxel									
				MDD lPCC	lMFG (-32, 38, 36)		–						~0.69	
					rMFG (32, 40, 40)		–						~0.66	
				MDD rPCC	lMFG (-26, 40, 20)		–						~0.54	
				H lPCC	lMFG (-36, 14, 58)		–						~0.63	
Schwarz et al. (2025) (54)		Functional Activation		Analysis	Conditions	Sample	Brain Region							
				Whole Brain	ATT>CON	1	R Superior Temporal Gyrus						~1.54**	
							L Superior Temporal Gyrus						~1.53**	
							R Middle Occipital Gyrus						~1.36**	
							R Inferior Frontal Gyrus, Triangular Part						~1.27**	
							L Middle Occipital Gyrus						~1.23**	
							L Lobule VIIB of Cerebellar Hemisphere						~1.17**	
							R Lobule VIII of Cerebellar Hemisphere						~1.12**	
							L Precentral Gyrus						~1.02**	
							R Lobule VI of Cerebellar Hemisphere						~0.99**	
							R Middle Frontal Gyrus						~0.94**	
							L Inferior Frontal Gyrus, Triangular Part						~0.94**	
							R Supplementary Motor Area						~0.87**	
Study	Measure	Analysis											d	
Schwarz et al. (2025) (54)	fMRI	Functional Activation		Analysis	Conditions	Sample	Brain Region							
				Whole Brain	ATT>CON	2	L Inferior Parietal Gyrus						~1.65**	
							R Inferior Parietal Gyrus						~1.49**	
							R Superior Temporal Gyrus						~1.43**	
							L Superior Temporal Gyrus						~1.32**	
							R Lobule VIII of Cerebellar Hemisphere						~1.31**	
							L Inferior Occipital Gyrus						~1.30**	
							L Precentral Gyrus						~1.22**	
				ROI		1	R Inferior Frontal Gyrus, Triangular Part						~1.13**	
							L Precentral Gyrus						~1.01**	
							R Middle Frontal Gyrus						~0.83**	
							L Middle Frontal Gyrus						~0.65*	
						2	L Precentral Gyrus						~1.22**	
							R Middle Frontal Gyrus						~0.85**	
				Whole Brain	ATT_(switch)_ > CON	1	L Inferior Frontal Gyrus, Triangular Part						~1.78**	
							R Middle Temporal Gyrus						~1.55**	
							L Lobule VI of Cerebellar Hemisphere						~1.49**	
							L Middle Occipital Gyrus						~1.49**	
							R Middle Occipital Gyrus						~1.43**	
							R Lobule VI of Cerebellar Hemisphere						~1.43**	
							R Lobule VIII of Cerebellar Hemisphere						~1.39**	
							L Supplementary Motor Area						~1.24**	
							L Lobule IV, V of Cerebellar Hemisphere						~0.99**	
							R Crus II of Cerebellar Hemisphere						~0.99**	
							R Middle Frontal Gyrus						~0.96**	
						2	L Inferior Parietal Gyrus						~1.97**	
							L Superior Temporal Gyrus						~1.66**	
							R Superior Temporal Gyrus						~1.58**	
							R Lobule VIII of Cerebellar Hemisphere						~1.50**	
							R Inferior Parietal Gyrus						~1.49**	
							R Inferior Occipital Gyrus						~1.44**	
Study	Measure	Analysis											d	
Schwarz et al. (2025) (54)	fMRI	Functional Activation		Analysis	Conditions	Sample	Brain Region							
				Whole Brain	ATT_(switch)_ > CON	2	L Insula						~1.44**	
							L Precentral Gyrus						~1.34**	
							L Middle Occipital Gyrus						~1.31**	
							R Insula						~1.29**	
							L Postcentral Gyrus						~1.24**	
							L Supplementary Motor Area						~1.19*	
							R Lobule VI of Cerebellar Hemisphere						~1.19*	
							R Inferior Frontal Gyrus, Triangular Part						~1.16*	
					ATT_(focus)_ > CON	1	L Superior Temporal Gyrus						~1.19**	
							R Inferior Occipital Gyrus						~1.07**	
							R Superior Temporal Gyrus						~1.06**	
							L Inferior Occipital Gyrus						~0.88*	
						2	R Inferior Occipital Gyrus						~1.23*	
					ATT_(switch)_ >ATT_(focus)_	1	L Precentral Gyrus						~1.21**	
							L Inferior Parietal Gyrus						~1.04**	
							L Supplementary Motor Area						~1.04**	
							L Fusiform Gyrus						~0.99**	
							L Middle Temporal Gyrus						~0.98**	
							R Calcarine Fissure and Surrounding Cortex						~0.96**	
							R Middle Frontal Gyrus						~0.92**	
							R Insula						~0.89**	
							R Lobule VI of Cerebellar Hemisphere						~0.89**	
							R Temporal Superior Gyrus						~0.89**	
							R Lobule VIII of Cerebellar Hemisphere						~0.88**	
							L Calcarine Fissure						~5.74*	
						2	*NO RESULTS*							
				ROI	ATT_(switch)_ > CON	1	L Rolandic Operculum						~1.50**	
							R Inferior Frontal Operculum						~1.33**	
							R Middle Frontal Gyrus						~0.92**	
						2	L Precentral Gyrus						~1.30**	
							R Inferior Frontal Gyrus, Triangular Part						~1.13**	
Study	Measure	Analysis											d	
Schwarz et al. (2025) (54)	fMRI	Functional Activation		Analysis	Conditions	Sample	Brain Region							
				ROI	ATT_(focus)_ > CON	1	R Middle Frontal Gyrus						~0.66*	
						2	L Inferior Frontal Gyrus, Triangular Part						~0.84*	
					ATT_(switch)_ > ATT_(focus)_	1	L Precentral Gyrus						~1.16**	
							R Middle Frontal Gyrus						~0.89**	
						2	L Inferior Frontal Operculum						~0.89*	
	
				Whole Brain	ATT > CON_(sound)_	1	R Middle OccipitalGyrus						~1.22**	
							L Middle Occipital Gyrus						~1.09**	
							R Inferior Frontal Gyrus, Triangular Part						~1.08**	
							R Middle Temporal Gyrus						~1.07**	
							L Inferior Frontal Gyrus, Triangular Part						~1.06**	
							L Middle Temporal Gyrus						~1.05**	
							L Lobule VIII of Cerebellar Hemisphere						~0.93**	
							L Lobule VI of Cerebellar Hemisphere						~0.93**	
							R Precentral Gyrus						~0.92**	
							R Lobule VIII of Cerebellar Hemisphere						~0.92**	
							Insula L						~0.89**	
							R Middle Frontal Gyrus						~0.88*	
						2	L Inferior Parietal Gyrus						~1.54**	
							R Inferior Parietal Gyrus						~0.99**	
							R Inferior Occipital Gyrus						~0.91*	
					ATT > CON_(white)_	1	L Superior Temporal Gyrus						~1.94**	
							R Superior Temporal Gyrus						~1.82**	
							R Inferior Occipital Gyrus						~1.25**	
							R Inferior Frontal Gyrus, Opercular Part						~1.23**	
							L Inferior Occipital Gyrus						~1.81**	
							L Inferior Frontal Gyrus, Triangular Part						~1.17**	
							R Inferior Parietal Gyrus						~1.16**	
Study	Measure	Analysis											d	
Schwarz et al. (2025) (54)	fMRI	Functional Activation		Analysis	Conditions	Sample	Brain Region							
				Whole Brain	ATT > CON_(white)_	1	L Lobule VIIB of Cerebellar Hemisphere						~1.04**	
							L Precentral Gyrus						~0.97**	
							L Middle Frontal Gyrus						~0.96**	
							L Supplementary Motor Area						~0.93**	
							L Cerebellum Crus 1						~0.93**	
							L Insula						~0.87*	
							R Cerebellum Crus 1						~0.85*	
					ATT > CON_(ATT)_	2	R Inferior Occipital Gyrus						~1.62**	
							R Supra Marginal Gyrus						~1.58**	
							L Inferior Parietal Gyrus						~1.46**	
							R Lobule VIII of Cerebellar Hemisphere						~1.41**	
							L Inferior Occipital Gyrus						~1.41**	
							R Inferior Frontal Gyrus, Triangular Part						~1.22*	
					CON_(sound)_ > CON_(white)_	1	L Superior Temporal Gyrus						~1.89**	
							R Superior Temporal Gyrus						~1.61**	
					CON_(ATT)_ > CON_(sound)_	2	*NO RESULTS*							
				ROI	ATT > CON_(sound)_	1	R Middle Temporal Gyrus						~0.95**	
							R Middle Temporal Gyrus						~0.78**	
							L Precentral Gyrus						~0.75**	
						2	L Precentral Gyrus						~1.03**	
							R Middle Frontal Gyrus						~0.86*	
							R Middle Frontal Gyrus						~0.84*	
					ATT > CON_(white)_	1	R Inferior Frontal Gyrus, Opercular Part						~1.19**	
							L Inferior Frontal Gyrus, Opercular Part						~1.06**	
							R Middle Frontal Gyrus						~0.82**	
					ATT > CON_(ATT)_	2	L Precentral Gyrus						~1.10**	
							R Inferior Frontal Operculum						~1.06**	
							L Inferior Frontal Gyrus, Triangular Part						~0.94*	
					CON_(sound)_ > CON_(white)_	1	L Inferior Frontal Gyrus, Opercular Part						~0.64*	
					CON_(ATT)_ > CON_(sound)_	2	*NO RESULTS*							

*p < 0.05; ** p < 0.01; ***p < 0.0125; EEG, Electroencephalography; EO, Eyes Open; EC, Eyes Closed; DA, Divided Attention; IR, Initial Resting; fNIRS, functional Near-Infrared Spectroscopy; ROI, Region of Interest; R, Right; L, Left; IFG, Inferior Frontal Gyrus; dlPFC, Dorsolateral Prefrontal Cortex; SAC, Somatosensory Association Cortex; fMRI, functional Magnetic Resonance Imaging; RUM, Rumination Induction Procedure; ATP, Abstract Thinking Procedure; FPN, Frontoparietal Network; DMN, Default Mode Network; DAN, Dorsal Attention Network; M_CAS, Main effect of CAS level; M_ATT, Main effect of intervention; Interact, Interaction of level of CAS and type of intervention; MDD, Major Depressive Disorder; H, Healthy sample. PCC, Posterior Cingulate Cortex; MFG, Middle Frontal Gyrus. CON, Control.

#### 4.2.1 Electroencephalography

Two studies used electroencephalography (EEG) to measure oscillatory activity during ATT practice. Knowles and Wells (28) found that one ATT dose revealed a distinct positive change in oscillatory signature, differing both in direction and pattern to a passive ATT control group. Greater positive frontoparietal changes, indicating neuronal synchrony, in the Anterior, then Midline and Posterior ROIs across both Alpha and Beta bands were found, coupled with minimal changes in Theta band activity across all ROIs. This was a markedly different neural signature to the control group who demonstrated negative changes across most ROIs for eyes open and approximately half for eyes closed. In contrast, Usui and colleagues (52) found no significant differences between the eight frequency bands analysed during an eyes open EEG recording task before and after 20-days of ATT practice. However, change indices for Alpha (1 and 2) were significant in the divided attention component. Alpha-1 activity was significantly reduced around the left orbital frontal gyrus (lOFG) and for Alpha-2, around the right inferior temporal gyrus (rITG), middle temporal cortex and fusiform.

#### 4.2.2 Functional near-infrared spectroscopy

One study (29) used functional near-infrared spectroscopy (fNIRS) to compare the haemodynamic changes of oxygenated (O_2_HB) and deoxygenated haemoglobin associated with an ATT-based neuroscientific paradigm across five ROIs: bilateral dorsolateral prefrontal cortex (dlPFC); bilateral inferior frontal gyrus (IFG) and somatosensory association cortex (SAC). In comparison to control condition, the ATT paradigm yielded significantly elevated blood oxygenation in the right IFG (*η^2^* = 0.087), right dlPFC (*η^2^* = 0.060) and SAC (*η^2^* = 0.078). Although there were no significant differences found between each of the three ATT components, effect sizes for the right IFG and SAC were typically larger in the attention switching condition (rIFG: *d* = 0.50; SAC *d* = 0.45), followed by the divided attention (rIFG: *d* = 0.33; SAC *d* = 0.43) and then selective attention (rIFG: *d* = 0.33; SAC *d* = 0.32) conditions. The right dlPFC yielded significantly different O_2_HB concentration in the attention switching condition compared to the control.

#### 4.2.3 Functional magnetic resonance imaging

Four studies used fMRI. Task-based fMRI findings by Jahn and colleagues (21) are reported in the CWST and EDP sections. Kowalski and colleagues (30) compared the effects of ATT in individuals with high and low levels of CAS. They found that following a rumination-induction procedure, a single ATT dose significantly decreased connectivity across frontoparietal networks (FPN) (81). Post-ATT, both high and low CAS groups (HCAS and LCAS, respectively) demonstrated decreased connectivity between the R-lateral prefrontal cortex (FPN) and L-inferior temporal gyrus; the R-lateral prefrontal cortex (FPN) and R-middle temporal gyrus; and the R-posterior parietal cortex (FPN) and R-middle temporal gyrus. Whereas decreased connectivity between the L posterior parietal cortex (FPN) and R-middle frontal gyrus was confined to the low CAS group only. Following an abstract-thinking procedure, ATT yielded significantly decreased connectivity between the precuneus cortex, part of the default mode network (DMN) (82) and L-lateral occipital cortex and precuneus cortex (DMN) and R-lateral occipital cortex with large and medium effect sizes, respectively. A significant decrease in resting state functional connectivity with a medium effect size was also found between the R-intraparietal sulcus, part of the DAN, and bilateral precuneus cortex (DMN).

Kowalski and colleagues (30) also used the inter-subject correlation (ISC) method to analyse the similarity of temporal synchronicity across participants. Except for the left frontal eye fields which only demonstrated a main effect of CAS-level, significant ATT-dependent effects were observed in all ROIs associated with DAN, FPN and DMN (*η^2^* = 0.22) with greater ISCs observed in the HCAS ATT group. Only a main effect of level of CAS was found across the PFN and DMN. The ISC in the precuneus cortex (DMN) was significantly higher in the HCAS ATT group, compared to the HCAS control and LCAS groups. In the left lateral PFC (FPN), an interaction of the level of CAS and intervention was found. The ISC of the HCAS ATT group was higher than both control groups but lower than the LCAS ATT group. Differences across ATT groups was also found between all ATT components, however *post-hoc* analyses suggested these differences were predominantly the result of the divided attention component across FPN and DAN networks (all *p* < 0.001) with variable findings between HCAS and LCAS groups. These findings demonstrate that ATT may differentially influence functional connectivity, dependent on the level (and possibly type) of CAS.

The above findings are partially supported by Müller and colleagues (53) who found significantly lower resting-state functional connectivity in the left posterior cingulate cortex (PCC) and bilateral middle frontal gyrus (MFG), regions belonging to DMN, within depressed individuals after 16 ATT doses. These findings differed to that of the heathy control group who showed significantly higher functional connectivity between left PCC and ipsilateral MFG post-ATT. These findings were, however, nonsignificant when comparing groups, over time. They found no significant ATT-dependent effects in brain regions associated with the salience (83), cortico-limbic (84) or executive control networks.

Recent work by Schwarz and colleagues (54), extended the work by Rosenbaum and colleagues (29), using an adapted neuroscientific ATT paradigm for fMRI to compare the differential effects of the selective attention and attentional switching ATT components. Across two independent samples, they found that ATT significantly enhanced activation in the FPN (inclusive of the bilateral PFC and intraparietal sulcus), in addition to the superior temporal, occipital gyrus and cerebellum. Attention switching elicited greater FPN activation than selective attention although both components additionally recruited temporal, occipital, and cerebellar brain regions in both samples, plus the bilateral insular in the second sample – differences attributed to the potentially greater demands that rapid attentional switching places on executive and linguistic-semantic processing. The selection of lower (CON_(white)_) and higher-level control conditions (CON_(sounds);_ CON_(ATT)_) in an attempt to isolate the active neural ingredients of ATT revealed significantly enhanced FPN activation during ATT compared to all control conditions. The ATT conditions were also associated with more effort and greater external attention focus compared to control conditions. Attention effort and also external-focusing effects of ATT were related to neural responses in the lateral PFC, supporting the use of internal-external ratings as a marker for ATT effects as per the original ATT protocol.

### 4.3 Conceptual synthesis by S-REF mechanisms

In this section we present a conceptual synthesis of the cognitive and neural findings in the context of the S-REF model. The goal was to assess if the pattern of findings is consistent with the causal mechanisms implicated in the model; ‘Threat Monitoring’, ‘Executive Control/Regulation’, and ‘Internally-focused CAS/Thinking Strategies (e.g. worry/rumination)’. To do so we assigned clusters of tasks/measures to the three mechanisms of the S-REF as follows:

#### 4.3.1 Threat monitoring

ATT-dependent effects on threat monitoring were assessed by synthesising findings from 11 studies that used the EST, EDP, DL, ANT (Alerting and Orienting indices), CTT1 and d2 TOA; tasks considered to reflect patterns of allocation, or otherwise processing biases that interact with metacognitive control. The threat monitoring component of the CAS refers to prolonged or intensified attention towards threat-related or emotionally-salient stimuli, perpetuating self-regulatory discrepancies (12, 13). ATT was designed to reduce persistent attention towards emotionally salient/threating information by improving ability to disengage and increase access to non-threat related information (3). ATT was associated with reduced hypervigilance and faster attentional disengagement from irrelevant and/or emotional stimuli towards relevant information on the EDP (19–21, 47); a finding that was not shown in the majority of non-threat related tasks (ANT (19, 25, 50), CTT1 (30) and d2 TOA (30, 41) with the exception of the DL (19, 20). Significant DL findings may, however, be attributed to general skill related improvements in auditory processing. Whilst there were no significant ATT-dependent EST effects identified (24, 48), perhaps due to suboptimal ATT implementation, the effect appeared to interact with conceptual elements of worry and rumination in a sample with elevated trait anxiety (24). Overall, these findings suggest that, consistent with its theoretical design, ATT selectively modifies biased attention towards emotion-related threat, enabling attention to be more easily redirected away from irrelevant and/or emotionally-salient stimuli - as opposed to specifically enhancing global attentional or emotional processing abilities.

#### 4.3.2 Executive control/regulation

ATT-dependent effects on executive control/regulation were assessed by synthesising behavioural findings (studies using the CWST, DNT, ACCE, ANT (executive index only) and CTT2 and neural evidence (EEG, fNIRS, and fMRI) from thirteen studies considered to reflect the S-REF model executive-level control system. ATT is intended to enhance higher-order cognitive control and therefore effects should be observed in tasks involving change in attention strategy, inhibition, and brain regions involved in higher-order control. Overall, these findings suggested that ATT consistently engages frontal brain regions/networks, supporting top-down cognitive control, flexible attentional shifting and modification of processing strategy dependent on task demands. ATT-dependent task performance effects were most consistent in tasks (e.g., CWST, DNT, ACCE and ANT) that purportedly tap into the underlying mechanisms of ATT – flexible and strategic disengagement from irrelevant stimuli towards more relevant stimuli (16, 22, 50, 51). These findings were also supported by neurocognitive effects across three different neural measures that consistently demonstrate ATT-dependent effects overlapping with brain regions responsible for executive control/regulatory processing (81, 85, 86). These include EEG, fNIRS and fMRI markers indicating ATT possibly induces changes in neural synchrony and increased activation and interconnectivity within the FPN (28, 30, 54), DAN (30), dlPFC (29, 52) and vlPFC (29). ATT-dependent effects also appear most pronounced across studies (16, 30, 50, 51) using samples/conditions relevant to clinical distress. Here potentially more doses provided more therapeutic benefit and those with higher CAS levels benefitted most from ATT training. These results align well with theoretical predictions about ATT counteracting core metacognitive processes contributing to the CAS.

#### 4.3.3 Internally-focused CAS/thinking strategies

Internally-focused, worry/rumination modes of thinking are part of the CAS and represent prolonged self-referential processing. We considered findings from mind-wandering as a proxy marker of the CAS and studies of activity in the DMN as mapping onto these S-REF mechanisms. ATT was designed to selectively target and reduce these CAS strategies, which should therefore be reflected in reduced activity in these tasks. This cluster consisted of three studies that were synthesised (30, 49, 53). ATT was associated with behavioural reductions in mind-wandering (considered a proxy marker of the CAS) as measured by the MWRA (49) and lower levels of spreading activation in the DMN in depressed patients (53) and healthy patients following a rumination-induction procedure (30). The DMN is associated with self-reflection, rumination and perseverative self-focused processing, hallmarks of the CAS and of reduced top-down cognitive control (87, 88). Together, these findings are consistent with the proposed effect of ATT in reducing CAS strategies and enabling disengagement from internal-focused attention and rumination based strategies.

## 5 Discussion

ATT is a metacognitive treatment technique designed to interrupt the CAS, enhance top-down flexible control of processing, and facilitate self-regulation (1). ATT has gained prominence as a possibly efficacious standalone transdiagnostic technique with promising effects across clinical samples. Elucidating whether the mechanisms underlying clinical effects are consistent with the S-REF model is essential to understanding and developing the technique. This is the first systematic review to systematically synthesise and evaluate the cognitive-attentional task performance and neurocognitive correlates of ATT. The review aimed to explore if there are specific or widespread effects, whether they are consistent across laboratory-based tasks/paradigms and neural measures and to understand if such effects are consistent with the mechanisms hypothesised in the S-REF model from which ATT is derived.

This review found reliable attention-task and neurocognitive effects associated with ATT detectable on a range (but not all) laboratory-based performance tasks and neural measures with small-to-large effect sizes; effects that are consistent with the attention disengagement and strategic executive control hypotheses of the S-REF model (12, 13). However, there is a high level of ambiguity and uncertainty within the literature which is compounded by wide task and method variation and inconsistency in ATT delivery making such conclusions tentative and preliminary.

ATT was designed to attenuate the CAS by reducing self-focused processing and enhancing attentional flexibility through the modification of higher-order metacognitive function (2). ATT appears to be associated with significant improvements in attentional flexibility through faster disengagement from task irrelevant and/or emotional stimuli, and reduced prioritisation of threat-based material across both visual and auditory modalities on some, but not all tasks. Nine out of fourteen tasks showed significant ATT-dependent performance effects in at least one study. These included two emotion-based tasks (EDP and ACCE) and seven tasks without an emotional component (CWST, DNT, ANT, DL, MWRA, Story Learning and Word-List Learning). Performance effects were most consistent on the EDP which showed moderate-to-large effects in four out of five studies, with higher doses increasing the magnitude of effect. The second most consistent effects were observed on the DL, with two out of three studies demonstrating moderate effect sizes. The largest effects, but least consistent overall, were shown in one study using the CWST and another using the ANT; though caution must be taken when interpreting these preliminary findings due to the absence of replication and limited evidence, at present.

Although inconsistent effects appeared between the two studies using the DNT, baseline ceiling effects on the DNT appeared to be a factor in one study. It is notable that the DNT studies were conducted on young children and rather than DNT performance, the primary outcome was a behavioural marker of delayed gratification (response inhibition). Whilst such measure is, strictly speaking, outside of the current review remit, the effect of ATT in improving delay of gratification seen in both studies also suggests that ATT effects are observable in behavioural measures of response inhibition in children, a marker of executive control.

The reliability of effects on the ACCE and MWRA currently remain unknown given that they were each only used by one study, and the ACCE study did not establish a true baseline. Nonetheless, detectable ATT-dependent effects across a range of tasks suggests that ATT may influence cognitive attentional task performance across different paradigms, and the data is clearly sufficient to warrant continued investigation. As most of these tasks (e.g., EST, EDP, DL, ANT CWST, DNT, ACCE and ANT) seem to measure the ability to override reflexive influences on processing and thus are pertinent to the purported top-down control mechanisms underpinning ATT, the findings appear to be consistent with ATT theoretical foundations and aims (12, 13). While it is important to acknowledge that most cognitive tasks involve both automatic and strategic processing, slower strategic adjustment is deemed essential to navigating the interference caused by exposure to conflicting and/or emotionally threatening information (63, 89–91) and the observed ATT-dependent task-related effects appear consistent with the modification of strategic self-regulation.

Some of the studies used tasks (e.g., N-Back, Story Learning and Word-List Learning) that are not directly relevant to the S-REF model. Story or word-list learning were used as memory-based measures and there is no basis in the theory for ATT influencing memory performance. Other tasks assessing memory performance (e.g., *N* Back) also did not detect an effect. Whilst mind wandering, as measured by the MWRA, may be considered a proxy marker of the CAS, the MWRA results may also not be highly relevant since it is not a recognised cognitive task, nor is it clear how it correlates with the other paradigms.

Inconsistencies were most pronounced in the EST. The variability may be accounted for by greater heterogeneity in task and methodological parameters, sample characteristics, and perhaps reflect level of ATT protocol adherence. Conceptually inappropriate and/or modified rationales and lack of/insufficient metalevel dialogue may have led to sub-optimal ATT implementation. Most therapeutic studies that failed to find an effect also report no attentional shift towards greater externality, a key indicator that ATT, as implemented, did not work as intended. The choice of experimental paradigm may have also influenced findings; the absence of effects on tasks such as the d2 TOA (except for 41) and CTT do not tap into the cognitive domains ATT is designed to modify (92, 93).

Evidence with small-to-large effect sizes appears to converge from the six studies using neural methodologies (EEG, fNIRS, fMRI), and which also support the preceding cognitive task findings. Together, they provide converging neural evidence that ATT modulates brain regions within cortical networks such as the CCN, FPN and DAN – areas responsible for top-down attentional flexibility and executive control (86). ATT’s neural signature also appears distinct from other attention-modification techniques supporting its ability to induce an alert attentional state through active engagement with ATT task instructions, but not through passive listening. For example, the absence of Theta-band activity and alpha symmetry found post-ATT contrasts to that of mindful meditation which yields Alpha, Beta and Theta-band activity and Alpha symmetry associated with parasympathetic activity and a relaxed drowsy state (94, 95).

Although neural findings are confined predominantly to healthy samples, specific regions such as the OFC and ACC associated with psychological disorder (96–98) appear to show diminished connectivity following ATT which could signal a potential neural mechanism of ATT’s clinical effects. ATT’s ability to enhance attentional disengagement from CAS-related processing may occur through inducing decreased communication between brain regions within the DMN, associated with self-referential, language, and emotional processing (99, 100). ATT-induced neural changes might also differ between clinical and non-clinical samples, depending on the level and type of CAS; perhaps those with higher levels of the CAS benefiting more from training.

It remains to be established if there is an optimal ATT dose and how this might differ across target populations. There is some indication that four doses may be optimal across healthy samples, but it remains unclear across clinical samples with variable results demonstrated between 7 to 13 doses. It also remains unclear whether it is the combination of components (i.e. the entire package) or individual components that might be associated with the effects observed. There is increasing evidence from two dismantling studies (29, 54) that the attentional switching component may be driving most of the associated effects. Two studies (30, 52) reported effects only in the divided attention component after employing the preceding ATT components, however this may be an effect caused by carry-over effects from the preceding ATT components.

## 6 Limitations

Low ATT adherence (including variations in ATT format and dose) and study quality/reporting issues (e.g., 18 out of 20 of the included studies were of moderate or weak study quality) were evident and may impact on consistency of the findings, as well as the validity and strength of the conclusions able to made from this review. Furthermore, ATT protocol deviations and suboptimal implementation could also have contaminated the integrity of the dataset available and consistency of findings within this review. Wide sample variations (e.g., children, adults, clinical vs non-clinical, multiple diagnostic categories, CAS levels), heterogeneity in choice and measurement of primary outcomes, and poor reporting standards in some studies also currently preclude meta-analytic approaches at scale. Meta-analytic approaches could in future provide improved precision (101). Whilst it is good practice to include heterogenous samples in evaluating the effect of a general technique, the high levels of heterogeneity across the included studies prevented completion of subgroup analyses per population and as such, caution should be taken when interpreting how ATT works in isolation for each specific subgroup included in this review. Caution should also be taken when interpreting the preliminary conclusions drawn from cognitive tasks represented by only one or two studies (e.g., CWST, ANT, DNT, ACCE, CTT, MWRA). Tasks such as the Stroop and EDP have been scrutinized for their psychometric properties and their limited applicability in capturing the dynamic context-dependent nature of attentional processes present within emotional disorders (84, 102–104). Whilst use of the S-REF framework might reduce such issues by conceptualising effects as more broadly indicative of a threat monitoring strategy in disorders, the role of wider metacognitive control mechanisms on attention allocation remain unspecified; to what extent can these particular tasks measure the higher order cognitive mechanisms that ATT is intended to modify?

## 7 Conclusion and future directions

The results of the systematic review are informative, demonstrating important cognitive and neural effects associated with ATT and highlighting areas for significantly improving the field of ATT research.

Taken overall, objective measures of cognitive and neural correlates show ATT effects most consistently clustering around the EDP task and brain regions associated with executive control and metacognition. Consistent effects have been observed in clinical, non-clinical and healthy samples, with provisional evidence of effects sensitive to levels of the CAS, as described in the S-REF model. A synthesis of the findings using the S-REF framework (the basis for ATT) shows findings consistent with the mechanisms of reduced threat monitoring, improved executive control, and improved disengagement from self-referential processing. ATT was found to be associated with attention task effects and modulation of brain regions within cortical networks involved in top-down attentional control and modulation of extended self-referential thinking. These core processes and control systems are central to the S-REF model and are what ATT was designed to modify, supporting the applicability and precision of this technique.

Despite these promising findings, a cautionary point should be made; task-specific cognitive effects are difficult to synthesise and interpret given uncertainties about the underlying processes that cognitive tasks are purported to assess and heterogeneity in the tasks, in scoring and reporting. Despite this the emotional dot-probe stands out as providing reliable findings. Neural results, on the other hand, show promising convergence indicating regional changes in EEG patterns, cerebral blood flow, and DMN connectivity consistent with the theoretical basis of ATT. Specifically, evidence supports the hypothesis that ATT is associated with modifying cognitive control and attentional flexibility (supported by higher-order metacognitive functions), and that the attention switching component of ATT may be of particular importance in this respect.

The review points to limitations in the literature, requiring future work to improve reporting of adherence and training in the ATT protocol, fidelity and integrity of the method, effective isolation of component effects, and the development of novel paradigms to assess S-REF mechanisms. In future studies we recommend: clearer specification of primary outcome measures and of research aims pertaining to whether ATT effects are being evaluated under full-treatment (requiring a minimum dose) or other “laboratory” conditions; clearer specification of the ATT version being used; reporting of ATT protocol adherence (including use of self-attention ratings and shaping of responses); and control of carry-over effects when attempting to isolate individual components. Individual differences in neurocognitive effects associated with ATT across clinical and sub-clinical populations using stimuli of self-relevant and disorder-specific nature should also be explored.

## Data Availability

The original contributions presented in the study are included in the article/**Supplementary Material**, further inquiries can be directed to the corresponding author/s.
