# Supplementary material for: Cognitive and neuropsychological correlates of the attention training technique: a systematic review and evidence synthesis
Source: Front Psychiatry. 2026 Jun 9;17:1766748. doi: 10.3389/fpsyt.2026.1766748 (PMC13288206; doi:10.3389/fpsyt.2026.1766748)
Supplement: Supplementary file 1 [file Table1.docx]

Supplementary Material

Supplementary Table 1. ATT Fidelity and Adherence Checklist

**The Attention Training Technique (Wells, 1990, Wells, 2009)**

Adherence and Fidelity Checklist

Authors:

**Title:**

|  | **Yes** | **Not Reported** | **Comments** |
| --- | --- | --- | --- |
| **Fidelity** | | | |
| Grounded in Wells’ metacognitive theory (e.g. reference to such; 1990, 2009) |  |  |  |
| Version referenced (1990, 2007, 2009)? |  |  |  |
| Permission obtained from originator? |  |  |  |
| Training received? (and type) |  |  |  |
| Method of delivery described ? (eg live, recorded) |  |  |  |
| Standalone technique? |  |  |  |
| **Adherence** | | | |
| Use of a credible and acceptable rationale? |  |  |  |
| Does it last for 10- 12 minutes? |  |  |  |
| Does it comprise of the three components (selective, switching, and divided attention)? |  |  |  |
| Are all three components completed within the one task? |  |  |  |
| Are there multiple sounds/spatial locations? |  |  |  |
| Has the task been modified in any way? |  |  |  |
| Was exploration and shaping of patients experience used? |  |  |  |
| Was practice/delivery embedded in metacognitive dialogue? |  |  |  |
| Was a credibility check completed? |  |  |  |
| Was a self-attention rating completed?  Was a criterion applied of minimum 2-point shift to externality? |  |  |  |
| Was homework practice encouraged? |  |  |  |
| Was homework encouraged to be completed when not in distress? |  |  |  |
| Did the rationale or homework encourage ATT to be used as a coping strategy? |  |  |  |
| Was homework encouraged to be used in combination with something else? |  |  |  |

**Other Comments**

Supplementary Table 2. Individual EPHPP Ratings

|  | 1. **Selection Bias** | | | 1. **Study Design** | | | | | 1. **Confounders** | | | 1. **Blinding** | | | 1. **Data Collection Methods** | | | 1. **Withdrawals /**   **Dropouts** | | | 1. **Intervention Integrity** | | | 1. **Analyses** | | | |
| --- | --- | --- | --- | --- | --- | --- | --- | --- | --- | --- | --- | --- | --- | --- | --- | --- | --- | --- | --- | --- | --- | --- | --- | --- | --- | --- | --- |
|  | **Q1** | **Q2** | **Tot** | **Q1** | **Q2** | **Q3** | **Q4** | **Tot** | **Q1** | **Q2** | **Tot** | **Q1** | **Q2** | **Tot** | **Q1** | **Q2** | **Tot** | **Q1** | **Q2** | **Tot** | **Q1** | **Q2** | **Q3** | **Q1** | **Q2** | **Q3** | **Q4** |
| **Sharpe et al. (2010) (53)** | 3 | 1 | 3 | 1 | Y | Y | Y | 1 | 1 | 1 | 1 | 1 | 2 | 2 | 1 | 1 | 1 | N/A | 1 | 1 | 3 | 2 | 5 | IND | GRP | 1 | 3 |
| **Levaux et al. (2011) (41)** | 3 | N/A | 3 | 7 | N | N/A | N/A | 3 | N/A | N/A | N/A | 1 | 1 | 3 | 3 | 3 | 3 | N/A | N/A | 2 | 1 | 2 | 4 | IND | IND | 1 | 3 |
| **Callinan et al. (2015)**  **(16)** | 3 | 0 | 3 | 1 | Y | Y | Y | 1 | 2 | N/A | 1 | 1 | 2 | 2 | 1 | 1 | 1 | N/A | 1 | 1 | 3 | 1 | 5 | IND | GRP | 1 | 3 |
| **Schwind et al. (2016) (52)** | 3 | 3 | 3 | 1 | Y | N | N/A | 1 | 2 | N/A | 1 | 1 | 2 | 2 | 1 | 1 | 1 | 1 | 1 | 1 | 3 | 2 | 4 | IND | GRP | 1 | 3 |
| **McEvoy et al. (2017) (24)** | 4 | 1 | 3 | 1 | Y | Y | Y | 1 | 1 | 3 | 3 | 3 | 2 | 2 | 1 | 1 | 1 | N/A | 1 | 1 | 3 | 1 | 5 | IND | GRP | 1 | 1 |
| **Taraban et al. (2017) (54)** | 3 | 1 | 3 | 1 | Y | Y | Y | 1 | 1 | 1 | 1 | 1 | 3 | 3 | 1 | 1 | 1 | N/A | 1 | 1 | 3 | 1 | 5 | IND | GRP | 1 | 3 |
| **Fergus & Hiraoka (2018) (48)** | 4 | 1 | 3 | 5 | N | N/A | N/A | 2 | N/A | N/A | N/A | 1 | 3 | 3 | 1 | 1 | 1 | 1 | 1 | 1 | 1 | 2 | 4 | N/A | IND | 1 | 3 |
| **Barth et al. (2019) (19)** | 3 | 1 | 3 | 1 | Y | N | N | 1 | 1 | 1 | 1 | 1 | 2 | 2 | 1 | 1 | 1 | N/A | 1 | 1 | 2 | 1 | 5 | IND | GRP | 1 | 3 |
| **Fernie et al. (2019) (47)** | 3 | 1 | 3 | 1 | Y | Y | Y | 1 | 2 | 1 | 1 | 1 | 2 | 2 | 1 | 1 | 1 | N/A | 1 | 1 | 3 | 1 | 5 | IND | GRP | 1 | 3 |
| **Heitland et al. (2020) (20)** | 3 | 1 | 3 | 1 | Y | N | N | 1 | 1 | 3 | 3 | 1 | 2 | 2 | 1 | 1 | 1 | N/A | 1 | 1 | 2 | 1 | 5 | IND | GRP | 1 | 3 |
| **Stewart et al. (2021)**  **(25)** | 4 | 1 | 3 | 1 | Y | Y | Y | 1 | 1 | 3 | 3 | 1 | 2 | 2 | 1 | 1 | 1 | 1 | 1 | 1 | 3 | 1 | 5 | IND | GRP | 1 | 3 |
| **Murray et al. (2016) (23)** | 2 | 1 | 2 | 1 | Y | Y | Y | 1 | 1 | 1 | 1 | 1 | 2 | 2 | 1 | 1 | 1 | 1 | 1 | 1 | 3 | 1 | 5 | GRP | GRP | 1 | 3 |
| **Murray et al. (2018)**  **(22)** | 2 | 1 | 2 | 1 | Y | Y | Y | 1 | 2 | N/A | 1 | 1 | 2 | 2 | 1 | 1 | 1 | 1 | 1 | 1 | 3 | 1 | 5 | GRP | GRP | 1 | 3 |
| **Knowles & Wells (2018) (28)** | 3 | 1 | 3 | 1 | Y | Y | Y | 1 | 2 | N/A | 1 | 1 | 2 | 2 | 1 | 1 | 1 | N/A | 1 | 1 | 3 | 1 | 5 | IND | IND | 1 | 3 |
| **Rosenbaum et al. (2018) (29)** | 3 | 5 | 3 | 1 | N | N | N/A | 1 | 1 | 1 | 1 | 1 | 2 | 2 | 1 | 1 | 1 | N/A | N/A | 2 | 1 | 1 | 5 | IND | GRP | 1 | 3 |
| **Kowalski et al. (2020)**  **(30)** | 2 | 3 | 3 | 1 | Y | Y | Y | 1 | 1 | 1 | 1 | 2 | 2 | 1 | 1 | 1 | 1 | 1 | 1 | 1 | 3 | 1 | 5 | IND | GRP | 1 | 3 |
| **Usui et al. (2022) (56)** | 3 | 1 | 3 | 5 | N | N/A | N/A | 2 | N/A | N/A | N/A | 3 | 3 | 2 | 1 | 1 | 1 | 3 | 3 | 3 | 1 | 1 | 5 | N/A | IND | 1 | 3 |
| **Jahn et al. (2023) (21)** | 3 | 1 | 3 | 1 | Y | Y | Y | 1 | 1 | 1 | 1 | 2 | 2 | 1 | 1 | 1 | 1 | 1 | 1 | 1 | 3 | 1 | 5 | IND | GRP | 1 | 3 |
| **Müller et al. (2025) (49)** | 3 | 1 | 3 | 1 | Y | Y | Y | 1 | 2 | N/A | 1 | 2 | 2 | 1 | 1 | 1 | 1 | N/A | 1 | 1 | 3 | 1 | 5 | IND | GRP | 1 | 3 |
| **Schwarz et al. (2025) (55)** | 3 | 1 | 3 | 2 | N | N | Y | 1 | N/A | N/A | N/A | 1 | 3 | 2 | 1 | 1 | 1 | N/A | 1 | 1 | 1 | 1 | 5 | IND | GRP | 1 | 3 |

Supplementary Table 3. Cognitive Attentional Task Descriptions by Study

| **Study** | **Task** | **Cognitive Domain** | **Delivery** | **Description** | **N Trials** | **Task Performance Calculation** |
| --- | --- | --- | --- | --- | --- | --- |
| **Fernie et al. (2019)**  **(47)** | **CWST** | EF: Response Inhibition | Computerised | Standard plus tactile inference condition (for increased perceptual load) on two of four CWST sessions. | 240 | RTs of correct responses plus Global Index (GI) score that represented the number of errors and speed of correct responses. |
| **Barth et al. (2019)**  **(19)** |  |  |  | Standard | 100 | Mean RTs of congruent trials (milliseconds) minus mean RTs of incongruent trials (milliseconds). Plus, contrast score of incongruent minus congruent RTs and incongruent RTs. |
| **Heitland et al. (2020)**  **(20)** |  |  |  |  |  |  |
| **Jahn et al., (2023)**  **(21)** |  |  |  |  |  |  |
| **Schwind et al. (2016)**  **(52)** | **EST** | EF: Response Inhibition (emotionally salient stimuli) | Computerised | Standard with threatening health and neutral words. | 240 | Difference between RTs for threat words and RTs for corresponding neutral words. |
| **McEvoy et al. (2017)**  **(24)** |  |  | Paper | Modified from Delis-Kaplan Executive Function System (D-KEFS) Colour-Word Interference Subtest (105) with contextual cue condition. | 200 | Total time of completion (seconds) and number of uncorrected errors for each trial with faster RTs (threat speed; neutral speed) and fewer errors (threat errors; neutral errors). |
| **Murray et al., (2016)**  **(23)** | **DNT** | EF: Verbal Response Inhibition | Paper | Standard | 16 | Difference between the number of errors at T1 and T2 |
| **Murray et al. (2018)**  **(22)** |  |  |  |  |  | Difference between the number of correct responses at T1 and T2 |
| **Sharpe et al. (2010)**  **(53)** | **EDP** | Attention/EF: Selective Attention / Disengagement  (emotionally salient stimuli) | Computerised | Pain-related word pairs from four pain categories. | 200 | Average congruent and incongruent RTs for each word-pair |
| **Barth et al. (2019)**  **(19)** |  |  |  | Negative word pairs from the ANGST database (106) | 100 | Mean neutral RTs - mean emotional RTs |
| **Heitland et al. (2020)**  **(20)** |  |  |  |  |  |  |
| **Stewart et al. (2021)**  **(25)** |  |  |  | Worry-based word pairs | 320 |  |
| **Jahn et al. (2023)**  **(21)** |  |  |  | Negative word pairs from the ANGST database (106). | 100 | Bias: incongruent mean RT – congruent mean RT  Orienting: neutral mean RT – congruent mean RT  Disengaging: incongruent mean RT – neutral mean RT  *As per* Salemink and colleagues (107). |
| **Callinan et al. (2015)**  **(16)** | **ACCE** | Attention/EF: Selective Attention / Disengagement  (emotionally salient stimuli) | Computerised | Modified version as per Johnson (63, 64) | 425 | Time (milliseconds) to disengage attention from an emotional face towards a neutral face. Two switch cost scores, emotion-neutral (EN) and neutral-emotion (NE) were calculated for each valence using median RTs. |
| **Fergus and Hiraoka (2018)**  **(48)** | **ANT** | Attention/EF | Computerised | Short version by Weaver and colleagues (109). | 124 | Alerting: mean RTs for the no cue condition - mean RTs for the double cue condition  Orienting: mean RTs in the centre cue condition - mean RTs from the spatial cue condition  Executive: mean RTs for the incongruent condition - mean RTs for the congruent condition |
| **Barth et al. (2019)**  **(19)** |  |  |  | Fan and colleagues (66) | 120 | Alerting: mean RT for the no cue condition - mean RT from the centre cue condition  Orienting: mean RTS in the centre cue condition - mean RTs from the spatial cue condition  Executive: mean RTS for the incongruent condition - mean RTs for the congruent condition |
| **Stewart et al. (2021)**  **(25)** |  |  |  | Fan and colleagues (65) - + neutral flanker | ? | Executive: mean RTS for the incongruent condition - mean RTs for the congruent condition |
| **Barth et al. (2019)**  **(19)** | DL | Attention (Auditory Selective) | Computerised | Standard (67) | 36 comb-inations | Weighted mean of all correct left and right ear RTs in the forced listening conditions; T1 – T2. |
| **Heitland et al. (2020)**  **(20)** |  |  |  |  |  |  |
| **Jahn et al. (2023)**  **(21)** |  |  |  |  |  | As above. Plus, single ear analyses. |
| **Rosenbaum et al. (2018)**  **(29)** | d2 ToA | Visual Sustained Attention/Processing Speed | Paper | Screening Only | | |
| **Kowalski et al. (2020)**  **(30)** |  |  |  | Polish adaptation (110) divided into the first and last seven rows, pseudorandomised for either pre- or post-ATT completion. | - | Total number characters processed = working attention speed  Percentage of errors = accuracy,  Total number of correctly cancelled letters minus the total number of incorrect cancellations = sustained attention/concentration |
| **Levaux et al. (2011)**  **(41)** |  |  |  | No description provided on test administration or scoring | - | Raw and standardised scores reported for processing speed, accuracy, global performance and concentration. |
| **Kowalski et al. (2020)**  **(30)** | CTT | Selective attention, task switching and visual processing speed | Paper | Polish adaptation (70) | - | Not reported. |
| **Barth et al. (2019)**  **(19)** | 3-Back | Working memory | Computerised | Braver and colleagues (111) | 150 | Means of correct target and non-target RTs **-** contrast scores between T1 and T2 |
| **Barth et al. (2019)**  **(19)** | 2-Back |  |  |  | 150 |  |
| **Heitland et al. (2020)**  **(20)** |  |  |  |  |  |  |
| **Jahn et al. (2023)**  **(21)** |  |  |  |  |  |  |
| **Taraban et al. (2017)**  **(54)** | MWRA | Mind wandering | Computerised | Franklin and colleagues (72) | 5129 words | The amount of time (RT) that lapsed between consecutive releases of the space bar. |
| **Levaux et al. (2011)**  **(41)** | Text Comprehension | Mind wandering / recognition memory | Paper | Lyubomirsky and colleagues (112) | 6 texts | Score on multiple choice test. The mind wandering frequency data from this task was not included in the review based on the subjectivity of scoring. |
|  | Story Learning | Immediate/delayed memory |  |  | 1 | ‘Recall as many details as possible’ – no further details provided on scoring. |
|  | Word-List Learning | Episodic memory |  | 15 abstract words | 5 | Recall on the first and fifth trial, total recall and delayed recall. Used as a control measure to exclude a general training effect of ATT. |
|  | Forward Span | Working memory |  | (74) | **-** | Not reported. Raw and standardised scores only. |
|  | Backward Span |  |  |  | **-** |  |
|  | Alpha Span |  |  | (75) | **-** |  |
|  | TAP | Flexibility |  | (76) | **-** |  |
|  | Go/No Go TAP | Inhibition |  |  | **-** |  |
|  | Incompatibility TAP |  |  |  | **-** |  |
|  | Hayling |  |  | (77) | **-** |  |
|  | Errand Test | Planning |  | (78) | **-** |  |
|  | RL/RI | Episodic verbal memory |  | (80) | **-** |  |

References

105. Delis, D. C., Kaplan, E., & Kramer, J. H. Delis-Kaplan executive function system: Examiner’s manual. San Antonio, TX: The Psychological Corporation. 2001.

106. Schmidtke DS, Schröder T, Jacobs AM,Conrad M. ANGST: Affective norms for German sentiment terms, derived from the affective norms for English words.Behav ResMethods. (2014)46:1108–18.doi:10.3758/s13428-013-0426-y

107. Salemink E, van den Hout M, Kindt M. Effects of positive interpretive bias modification in highly anxious individuals. J Anxiety Disord. (2009) 23:676–83. doi:10.1016/j.janxdis.2009.02.006

108. Posner MI, Rothbart MK.Fifty years integrating neurobiology and psychology to study attention. BiolPsychol. (2023) 180: 108574. doi:10.1016/j.biopsycho.2023.108574

109.Weaver B, Bédard M, McAuliffe J. Evaluation of a10-minute version of the attention network test. ClinNeuropsychol. (2013) 27:1281–99. doi: 10.1080/ 13854046.2013.851741

110. Dajek ER. Polska standaryzacja Testud 2, testu badania uwagi R. Brickenkampa. In:ERDA(2003).

111. Braver TS, Cohen JD, Nystrom LE, Jonides J, Smith EE, Noll DC. A parametric study of prefrontal cortex involvement in human working memory. NeuroImage. (1997)5:49–62.Available online at: https://www.sciencedirect.com/science/article/abs/ pii/S1053811996902475.

112. Lyubomirsky S. Dysphoric rumination impairs concentration on academic tasks. CognTherRes. (2003)27:309–30. doi: 10.1006/nimg.1996.0247
